# Standards for model-based early bactericidal activity analysis and sample size determination in tuberculosis drug development

**DOI:** 10.3389/fphar.2023.1150243

**Published:** 2023-04-13

**Authors:** Laurynas Mockeliunas, Alan Faraj, Rob C. van Wijk, Caryn M. Upton, Gerben van den Hoogen, Andreas H. Diacon, Ulrika S. H. Simonsson

**Affiliations:** ^1^ Department of Pharmaceutical Biosciences, Uppsala University, Uppsala, Sweden; ^2^ TASK, Cape Town, South Africa

**Keywords:** tuberculosis, early bactericidal activity, sample size, pharmacometrics, model-based analysis

## Abstract

**Background:** A critical step in tuberculosis (TB) drug development is the Phase 2a early bactericidal activity (EBA) study which informs if a new drug or treatment has short-term activity in humans. The aim of this work was to present a standardized pharmacometric model-based early bactericidal activity analysis workflow and determine sample sizes needed to detect early bactericidal activity or a difference between treatment arms.

**Methods:** Seven different steps were identified and developed for a standardized pharmacometric model-based early bactericidal activity analysis approach. Non-linear mixed effects modeling was applied and different scenarios were explored for the sample size calculations. The sample sizes needed to detect early bactericidal activity given different TTP slopes and associated variability was assessed. In addition, the sample sizes needed to detect effect differences between two treatments given the impact of different TTP slopes, variability in TTP slope and effect differences were evaluated.

**Results:** The presented early bactericidal activity analysis approach incorporates estimate of early bactericidal activity with uncertainty through the model-based estimate of TTP slope, variability in TTP slope, impact of covariates and pharmacokinetics on drug efficacy. Further it allows for treatment comparison or dose optimization in Phase 2a. To detect early bactericidal activity with 80% power and at a 5% significance level, 13 and 8 participants/arm were required for a treatment with a TTP-EBA_0-14_ as low as 11 h when accounting for variability in pharmacokinetics and when variability in TTP slope was 104% [coefficient of variation (CV)] and 22%, respectively. Higher sample sizes are required for smaller early bactericidal activity and when pharmacokinetics is not accounted for. Based on sample size determinations to detect a difference between two groups, TTP slope, variability in TTP slope and effect difference between two treatment arms needs to be considered.

**Conclusion:** In conclusion, a robust standardized pharmacometric model-based EBA analysis approach was established in close collaboration between microbiologists, clinicians and pharmacometricians. The work illustrates the importance of accounting for covariates and drug exposure in EBA analysis in order to increase the power of detecting early bactericidal activity for a single treatment arm as well as differences in EBA between treatments arms in Phase 2a trials of TB drug development.

## 1 Introduction

*Mycobacterium tuberculosis* (Mtb) is the pathogenic bacteria that causes tuberculosis (TB), one of the leading causes of death from an infectious disease (World Health Organization, 2021). While progress in TB treatment shortening has accelerated in recent years, with the success of the Nix-TB trial showing that 6-month treatment is possible for drug-resistant TB (Conradie et al., 2020) and drug-susceptible TB treatment can be shortened to 4-month with rifapentine-moxifloxacin regimen (Dorman et al., 2021), TB remains a global problem. Many novel drugs are in the TB drug development pipeline, promising a potential trove of new medications in the future (Working Group on New TB Drugs, 2022).

One of the critical steps in TB drug development is a Phase 2a early bactericidal activity (EBA) study. An EBA study is an established method to provide clinical proof of concept for antimicrobial drugs under investigation (Jindani et al., 1980; Donald and Diacon, 2008; Food and Drug Administration, 2013; European Medicines Agency, 2017). EBA studies investigate if a drug is active in patients and represents a major milestone that unlocks further investment for clinical development. Most of the drugs active against Mtb show activity in EBA trials with very few exceptions, e.g., clofazimine which has shown no EBA *in vitro*, *in vivo* and patients but is believed to have an activity against persisting bacteria (Ammerman et al., 2017; Faraj et al., 2020). In addition to proof of concept, the EBA trials provide the opportunity to study safety, tolerability and pharmacokinetics (PK) in patients. Depending on the EBA trial design, the relationship between activity and dose can be studied as well as the activity of different treatments can be compared which can guide further clinical development. Usually, EBA studies are conducted in a carefully controlled setting with a small number of patients per group (12–15 participants) for up to 14 days, and include sputum sample collection, PK sampling, and intensive safety assessments.

EBA is quantified by measurement of the viable mycobacterial load in overnight-collected sputum samples over time using colony forming units (CFU) of Mtb (measured in log10 CFU/mL sputum) and/or time-to-positivity (TTP) in liquid culture (measured in hours). The correlation between changes in TTP and CFU varies over time, which is due to that they likely reflect slightly different processes (Diacon et al., 2012; Bowness et al., 2015; Ayoun Alsoud et al., 2022). CFU measures the quantity of viable mycobacteria regardless of the speed of growth, while TTP measures the consumption of critical ingredients in a closed liquid culture system, which is influenced by both the quantity and metabolic activity of the growing mycobacteria (Alffenaar et al., 2022). These assays are inherently variable and should be performed with at least two replicates, where overnight-collected sputum samples from each day are subsequently divided to multiple replicates. In addition to established culture-based biomarkers like CFU and TTP, new biomarkers are currently being developed, which could complement or replace existing ones in the future. With all of the promising progress in the TB drug pipeline, it is critical to have a robust and standardized approach to analyzing EBA trial data.

Approaches present for EBA analysis can be categorized into i) traditional non-model-based EBA analysis, ii) traditional model-based EBA analysis, and iii) pharmacometric model-based EBA analysis. The first paper presenting an EBA trial and analysis was a traditional non-model-based analysis presenting the results of a 14-day duration trial (Jindani et al., 1980). The differences in CFU, expressed as log_10_ CFU between day 0 and day 2, day 2 and day 14, as well as day 0 and day 14, were derived on the individual level. To compare different treatments, the mean fall in CFU was calculated using ANOVA. Since then, the traditional non-model-based EBA analysis approach was extended to analyze both CFU and TTP biomarkers (Eq. 1), where EBA ( $\left.{EBA}_{t_{1}-t_{2}}\right)$ is expressed as a difference between the biomarker observations taken at the second time point ( $\left.{Obs}_{t_{2}}\right)$ and the observation taken at first time point t_1_ ( $\left.{Obs}_{t_{1}}\right)$ divided by the time interval (t_2_ minus t_1_) (Jindani et al., 1980). This approach provides a model-free estimate of EBA, which can later be used for the comparison of different treatments.

$$\begin{array}{c} {EBA}_{t_{1}-t_{2}}=\frac{{Obs}_{t_{2}}-{Obs}_{t_{1}}}{t_{2}-t_{1}} \end{array}$$ (1)

Non-model-based EBA approach is frequently substituted by the traditional model-based EBA approach. Here, EBA is estimated using a function, i.e., linear, bi-linear, etc. (Eq. 2), as shown in the paper by Jindani et al. (Jindani et al., 2003):

$$\begin{array}{c} {EBA}_{t_{1}-t_{2}}=\frac{f\left(t_{2}\right)-f\left(t_{1}\right)}{t_{2}-t_{1}} \end{array}$$ (2)

$f\left(t\right)$ represents a fitted regression function to the biomarker data, and $f\left(t_{n}\right)$ represents a biomarker value for day *n* derived using a fitted function.

In the traditional model-based EBA analysis approach, data from all timepoints is used to describe the change in biomarker (CFU and/or TTP) over time using linear, bi-linear or multiple linear regression models. In the case of a linear model, the biomarker gradually changes over time with a constant slope value. In the case of bi-linear regression, two distinct phases of change in load are observable (Diacon et al., 2013).

An extension to the traditional model-based EBA analysis is pharmacometric model-based analysis. A pharmacometric approach is built on non-linear mixed-effects modeling. Usually, these models are composed of structural, stochastic, and covariate sub-models. The structural sub-model defines the underlying change in the biomarker over time and consists of parameters called fixed effects which represent the change in biomarker over time in a “typical patient”. The stochastic sub-model includes random effect parameters which describe inter-individual variability (IIV) and residual unexplained variability (RUV). IIV is related to between patient variability, and RUV is due to variation in each observation from the model prediction due to unexplained factors such as imprecision in the biomarker assay, sample collection and handling, and model misspecification. The addition of a covariate sub-model containing information about influential factors on variability in data can reduce unexplained variability in the model and can also lead to treatment optimization in sub-populations and improved EBA characterization. In a pharmacometric model-based analysis, drug exposure as a covariate is often considered in the model to explain variability in response, which enables clinical trial simulations using dosing regimens not used in the trial. The pharmacometric model-based approach has been applied to multiple Phase 2a trials in TB, including rifampicin (Svensson and Simonsson, 2016), clofazimine (Faraj et al., 2020) and meropenem-containing treatments (De Jager et al., 2022). In addition, model-based pharmacokinetic-pharmacodynamic (PK-PD) approaches have shown to have higher statistical power compared to other analysis approaches (Svensson et al., 2017).

The aim of this work was to present a standardized pharmacometric model-based EBA analysis approach. In addition, the aim was to perform sample size determinations for detecting EBA or a difference between treatment groups by employing a pharmacometric model-based EBA analysis approach.

## 2 Materials and methods

### 2.1 Standardized pharmacometric model-based early bactericidal activity analysis approach

**FIGURE 1:**
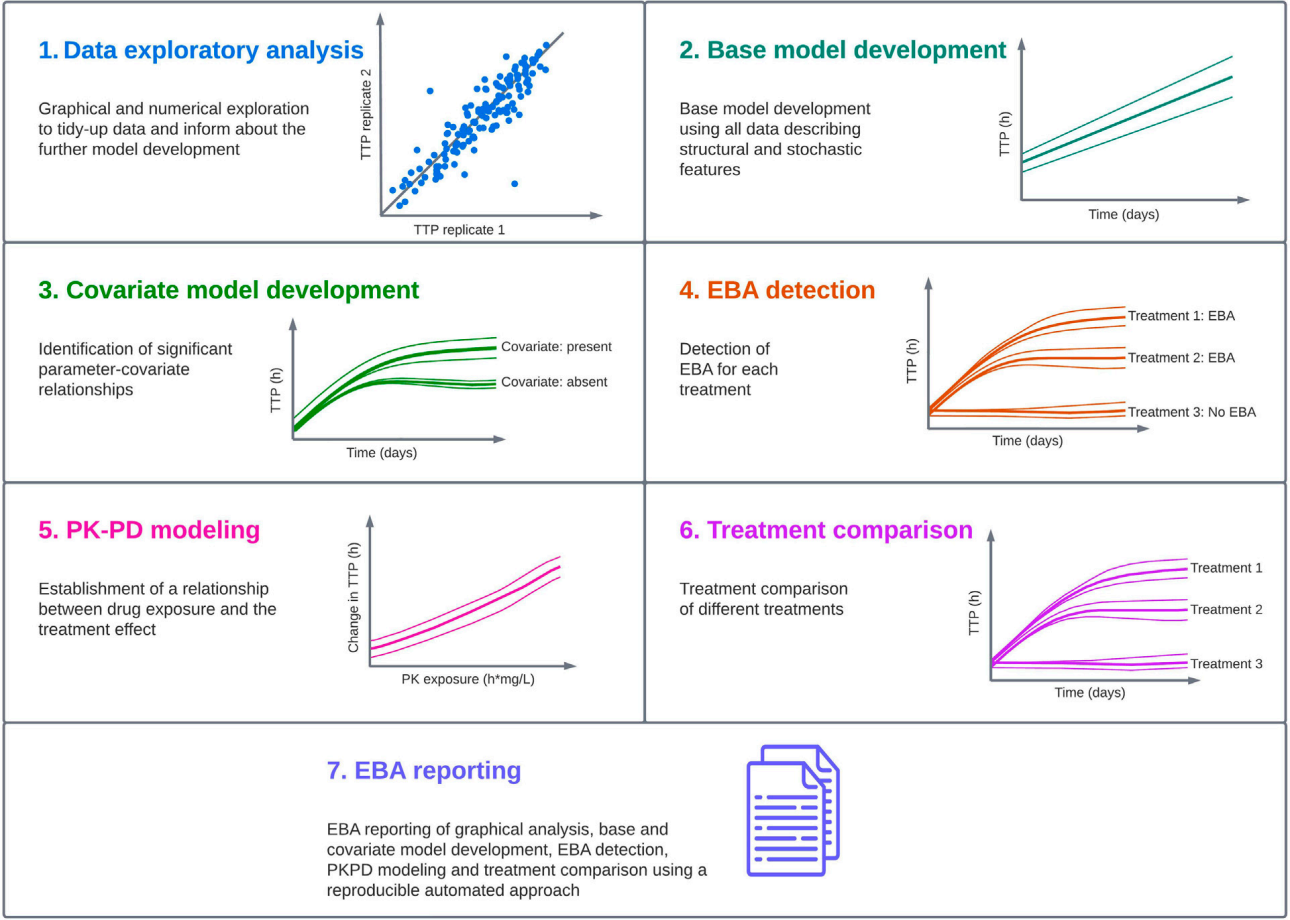
Standardized pharmacometric model-based early bactericidal activity (EBA) analysis approach was established in close collaboration between pharmacometricians, clinicians, microbiologists and data managers. In the pharmacometric model-based EBA analysis approach, the first step is to perform data exploratory analysis to familiarize with the data and identify observations that could affect the analysis. In the next step, modeling is started with a base model development, and this model is used in the covariate model building to identify statistically significant parameter-covariate relationships. Afterwards, EBA detection is performed to identify treatments showing EBA. In the next step, if drug exposure information is available, pharmacokinetic-pharmacodynamic (PK-PD) modeling should be performed. This is followed by treatment comparison if multiple treatments are present in the study. In the last step, EBA reporting is done using an automated and reproducible approach to ensure consistency, and the predictions of EBA on typical and individual levels are presented.

In order to characterize a standardized pharmacometric model-based EBA analysis approach, seven critical steps were identified; exploratory data analysis, base model development, covariate analysis, EBA detection, PK-PD modeling, treatment comparison and reporting (Figure 1). These steps are in line with the guidelines from the United States Food and Drug Administration (FDA) and the European Medicine Agency (EMA) for conducting and reporting pharmacometric analysis (European Medicines Agency, 2008; Food and Drug Administration, 2022). Close collaboration is required to facilitate an EBA analysis that supports decision making in the clinical development of new TB drugs. This requires efficient, smooth and optimal collaboration between the pharmacometricians performing data analysis and modeling, clinicians responsible for the clinical trial and patients, microbiologists analyzing the samples and quantifying the bacterial burden, and data managers responsible for data handling.

Below, each step of the standardized pharmacometric model-based EBA approach is presented for the analysis of the biomarker TTP. This approach is easily extendable to other EBA biomarkers such as CFU but also to future biomarkers with longitudinal decline or increase over time.

#### 2.1.1 Simulated early bactericidal activity data

In order to visualize every step of the pharmacometric model-based EBA modeling workflow, a 14-day EBA trial was simulated using a simulation model composed of the final parameter estimates from two previous TTP-EBA models based on two meropenem-containing treatments (Supplementary Table S1) from De Jager et al. (De Jager et al., 2022) and unpublished data (ClinicalTrials.gov Identifier: NCT04629378). In total, longitudinal TTP data in 30 participants, divided into two different treatment arms (Arm A and B), were simulated. Four different covariates were simulated; age, sex, cavity extent at baseline and meropenem area under the concentration *versus* time curve from time zero to infinity (AUC_0-inf_). Cavity extent at baseline had three categories; no cavities, cavities <4 cm, and cavities ≥4 cm. Five participants per each cavity category per treatment were simulated. Meropenem AUC_0-inf_ at day 14 were sampled from a normal distribution with a mean of 640 h mg/L and a standard deviation of 86 h mg/L (unpublished data, ClinicalTrials.gov Identifier: NCT04629378). No difference in AUC_0-inf_ between the two treatment groups was assumed. Equal distribution of sex was simulated in the trial (15 males, 15 females). Age distribution was simulated from a normal truncated distribution with a mean of 33 years, a lower boundary of 18 years, an upper boundary of 60 years and a standard deviation of 13 years. The covariate relationships included in the simulations were cavity on TTP baseline and AUC_0-inf_ on TTP slope (Supplementary Table S1). One of the arms (Arm A) were simulated to receive a meropenem containing regimen consisting of 2 g meropenem, 500 mg amoxicillin and 125 mg clavulanate thrice daily on days 1–14 (De Jager et al., 2022). The second arm (Arm B) was simulated to receive a treatment of 6 g meropenem, 2000 mg amoxicillin, 62.5 mg clavulanate and 400 mg bedaquiline once daily on days 1–14 (unpublished data, ClinicalTrials.gov Identifier: NCT04629378). To resemble a realistic EBA study, a proportion of the simulated observations were randomly assigned to the following status; negative at day 42 (2%), missing (2%), contaminated (2%) and not done (2%).

The simulation model consisted of a mono-exponential model with the following structure:

$$\begin{array}{c} TTP=e^{A_{mono}}∙e^{\alpha_{mono}∙time} \end{array}$$ (3)

where *A* is the intercept (TTP baseline), and *α* is the slope of the *TTP* decline over time. A proportional error model was used with both common and replicate-specific error terms for each replicate (Supplementary Table S1).

#### 2.1.2 Data exploratory analysis

Exploratory analysis of clinical trial data is the first step in model development and it should be performed both graphically and numerically (European Medicines Agency, 2008; Food and Drug Administration, 2022). It should be performed before model development commences and be reviewed by clinicians and microbiologists responsible for the trial conduct and data generated. Discussion around the outcomes of the graphical and numerical exploratory analysis should lead to consensus on exclusion of outlying observations or participants.

Collaborative expertise is vital for a good understanding of the data as well as for adequate quality of the analysis. At this stage, pharmacometricians together with clinicians identify observations in the graphical and numerical analysis, which should be further investigated and potentially excluded from further analysis. Microbiologists investigate observations in question and provide any additional information on the experiments, if available, which could strengthen the grounds for data exclusion.

Graphically, it is important to identify extreme values that could potentially interfere with the analysis, and help familiarize with the data to facilitate model development. Replicate-versus-replicate figures, displaying one replicate on the *x*-axis and the other replicate on the *y*-axis, are expected to show a scatter around the line of identity. Clear outlying datapoints should be reviewed carefully. Biomarker-versus-biomarker plot (showing one biomarker plotted *versus* another biomarker) can be created if multiple biomarkers were used to evaluate the bacterial burden over time, and datapoints outside of the scatter should be reviewed carefully. Biomarker-over-time curves for the population, either as scatter plots or based on summary statistics, will inform on the shape of the bacterial burden over time, and which model functions to evaluate. Individual bacterial burden over time, preferably with adjacent panels for different biomarkers of comparison, can support a review of the outlying datapoints from the replicate-versus-replicate and/or biomarker-versus-biomarker figures. To minimize bias, masking the group allocation and randomization of individuals in graphs should be considered. In addition, baseline bacterial burden can be shown stratified over covariates (boxplots for categorical covariates, scatter plot for continuous covariates), as well as a biomarker-over-time stratified over covariates (categorization of continuous covariates), and this can inform on the covariate relationships to test.

Numerically, the exploratory analysis should help with familiarization with data and numeric comparison between treatment groups. Summary statistics (median, interquartile range, mean, standard deviation) of the bacterial burden will provide initial estimates for baseline population parameters as well as inter-individual and residual variance parameters. Summary statistics on demographics and additional disease biomarkers (e.g., imaging-based markers such as cavity extent) can be used to check randomization processes as these variables should be similarly distributed across the study arms. These may also suggest covariate relationships to include in the model development strategy. Measures of exposure should also be numerically explored overall and per arm through summary statistics on secondary PK parameters such as area under the curve (AUC), maximum concentration (C_max_)_,_ and minimum concentration (C_min_). The proportion of missing data, either because of the contaminated sample, no result from the assay, sample not taken/performed, or other reasons, should be assessed for unequal distribution across arms as this otherwise may bias analysis outcomes. The number of censored observations, either 0 log_10_ CFU/mL sputum for CFU or the negative liquid culture at day 42 for TTP are in general indicative of the presence of EBA and favorable outcome.

#### 2.1.3 Base model development

For the structural model, both mono- and bi-exponential models should be evaluated for each biomarker; TTP (Eqs. 4, 5 and CFU Eqs. 6, 7).

$$\begin{array}{c} {TTP}_{pred}=e^{A_{mono}}∙e^{\alpha_{mono}∙time} \end{array}$$ (4)

$$\begin{array}{c} {TTP}_{pred}=e^{A_{bi}}∙e^{\alpha_{bi}∙time}+e^{B_{bi}}∙e^{\beta_{bi}∙time} \end{array}$$ (5)

$$\begin{array}{c} {\mathrm{Log}}_{10}{CFU}_{pred}=\log_{10}\left(e^{A_{mono}}∙e^{{-\alpha }_{mono}∙time}\right) \end{array}$$ (6)

$$\begin{array}{c} {\mathrm{Log}}_{10}{CFU}_{pred}=\log_{10}\left(e^{A_{bi}}∙e^{{-\alpha }_{bi}∙time}+e^{B_{bi}}∙e^{{-\beta }_{bi}∙time}\right) \end{array}$$ (7)

where *A*_*mono*_ and *A*_*bi*_ are the baseline for mono- and bi-exponential models respectively, $B_{bi}$ is the intercept for the second slope in a bi-exponential model, $\alpha_{mono}$ , $\alpha_{bi}$ and $\beta_{bi}$ are the slopes of the curve. Log_10_ is the logarithm with base 10, *e* is the exponential function. ${CFU}_{pred}$ is the predicted CFU (expressed as log_10_ CFU/mL sputum), ${TTP}_{pred}$ is the predicted TTP (expressed as hours). The magnitude of the CFU observations requires the data to be modelled on the log scale where the range of TTP is smaller and therefore indicates that the TTP data can be modelled on the natural logarithm scale.

Generally, these models are sufficient to describe the data, but more advanced non-linear models such as those including time-dependent functions could be considered if model diagnostics for the mono- and bi-exponential models show insufficient model performance (De Jager et al., 2022). For CFU modeling, negative slope(s) should be evaluated, while for TTP modeling positive slope(s) are appropriate.

The stochastic model captures IIV and RUV IIV terms can be evaluated on the population parameters (e.g., intercept, slope) as in Eq. 8, resulting in a lognormal distribution with a lower bound of zero.

$$\begin{array}{c} {Parameter}_{individual}={TV}_{parameter}∙e^{\eta } \end{array}$$ (8)

where TV is the typical value and *η* is drawn from a normal distribution with a mean of zero and standard deviation *ω*. Other distributions *via* transformations can be considered if the diagnostics of the *η* distribution indicate it.

The RUV model should capture the residuals in a homoscedastic manner across time and predictions. For log-transformed data such as CFU, an additive residual error model on the log-transformed scale is appropriate as it approximates a proportional error model on the untransformed scale. For untransformed data, an additive (Eq. 9), proportional (Eq. 10), or combination of additive and proportional error (Eq. 11) model should be evaluated. In the case of multiple replicate measurements from the same sample, a RUV model with combined and separate error terms can be utilized to quantify both the shared part of the residual noise from the single sample as well as the separate part of the residual noise from the assay replicate (Eq. 12), or by quantifying the correlation of the level 2 (L2) random effect in non-linear mixed effects modeling algorithms (Karlsson et al., 1995).

$$\begin{array}{c} Y=IPRED+\varepsilon_{a} \end{array}$$ (9)

$$\begin{array}{c} Y=IPRED∙\left(1+\varepsilon_{p}\right) \end{array}$$ (10)

$$\begin{array}{c} Y=IPRED∙\left(1+\varepsilon_{p}\right)+\varepsilon_{a} \end{array}$$ (11)

$$\begin{array}{c} Y=IPRED+\varepsilon_{1}+\varepsilon_{2}\\ IF\left(REP.EQ.2\right)\;Y=IPRED+\varepsilon_{1}+\varepsilon_{3} \end{array}$$ (12)

in which Y is the observation, IPRED is the individual prediction, REP is the replicate (here numbered either 1 or 2), $\varepsilon_{a}$ is the additive error term, $\varepsilon_{p}$ is the proportional error term, $\varepsilon_{1}$ is the shared error term, and $\varepsilon_{2}$ and $\varepsilon_{3}$ are the replicate-specific error terms, all of which are randomly drawn from a normal distribution with mean 0 and standard deviation σ.

#### 2.1.4 Covariate model development

An important step in the model-building process is to identify significant parameter-covariate relationships. The overall goal with covariates is to describe and explain observed between patient variability, which in turn will support predicting EBA for relevant subpopulations but most importantly, by explaining between patient variability, the power to detect EBA or differentiate EBA between drugs or treatments, will increase.

In the Phase 2a trial setting, a set of different covariates is collected. A selected list of covariates should be identified based on correlations plots of covariates and individual TTP slope in addition to experience based on historical EBA trials. It is important to pay attention to shrinkage when evaluating plots based on Bayes estimates (Savic and Karlsson, 2009). Covariates which should be evaluated on biomarker baseline (and additional intercepts) are; sex, body mass index (BMI), HIV status, ethnicity and age. Covariates to be evaluated on change in bacterial load over time (TTP and/or CFU slope) are; sex, presence of cavity, grade of cavitation and initial bacterial load. Additional covariates, like drug susceptibility, presence of concomitant diseases/medications and others can be evaluated, if data is available and there are grounds for the covariate to be evaluated. For simplicity in this work, we only illustrate graphical analysis and covariate analysis with sex, age and cavity extent.

Covariate analysis can be performed using different methods. One of the most common methods is stepwise covariate modeling (SCM) (Lindbom et al., 2005). An inclusion criterion for covariates of *p* ≤ 0.05 followed by a backwards deletion criteria of *p* ≤ 0.01 are suggested to be used. Power functions of relationships between model parameter and covariates are evaluated. Other parametrizations of covariate relationships may be considered if indicated by the data. Categorical covariate-parameter relationships are implemented as a fractional difference to the most common category.

#### 2.1.5 Early bactericidal activity detection

After the covariate model has been established, a formal statistical testing of EBA can be done for each of the different arms in the EBA trial, one by one. An exception could be for control arms which usually have lower sample size than the experimental arms. Typical parameters (excluding baseline bacterial load) will be fixed to 0 and compared to a model estimating the respective parameters, for each treatment one by one in order to confirm EBA. Statistical significance, often at a 5% significance level considering the degrees of freedom, is tested using the likelihood ratio test between the models. Treatment arms for which the TTP (or CFU) slope(s) are statistically significant are considered to have confirmed EBA at the sample size in the trial. Similar, treatment arms for which the TTP (or CFU) slope(s) are statistically not significant are considered to have no EBA at the sample size in the trial. For treatment arms with no identified EBA, the TTP or CFU slope(s) are fixed to zero in the further model development.

#### 2.1.6 Pharmacokinetic-pharmacodynamic modeling

PK-PD modeling allows for accounting for between patient variability in drug exposure which may be a reason for difference in EBA between patients. PK-PD modeling can be done by connecting a population PK model to the EBA model and where predicted concentration over time drives the PK-PD relationship (Zhang et al., 2003). An alternative is to use predicted drug exposure indices (AUC and/or C_max_) as a covariate on the slope(s) in the EBA model. AUC and C_max_ can be predicted from population PK model or obtained from non-compartmental analysis (NCA) which can be obtained if the PK sampling is rich and well designed. Different PK-PD relationships on the different EBA slope(s) are evaluated such as linear and non-linear relationships. If between-subject variability in EBA slope(s) can be described by PK, this reduce the unexplained between patient variability in EBA which thereby increases the power to detect EBA.

After the PK-PD relationship was established, it can be visualized by performing simulations using the final model, i.e., by using sampling importance resampling (SIR) to derive the uncertainty around the parameters, followed by using the output from SIR in stochastic simulations and estimations to derive the relationship on the typical individual level. In this case, only the typical values are unfixed, and remaining parameters, like IIV and RUV are fixed to zero.

#### 2.1.7 Early bactericidal activity comparison

If multiple treatment arms are included in the EBA trial design, arms that have shown statistically significant EBA, will be taken forward to EBA comparison evaluation to support the comparison of treatment arms. The final covariate EBA model is used to evaluate differences in regimen efficacy. During the evaluation of treatment efficacy, included IIVs, except IIV on baseline bacterial load, will initially be fixed to zero. For treatment arms with no EBA, the TTP slope will be zero. Treatment arms that have shown EBA in the earlier step, are initially defined as having the same TTP (and/or CFU) slope(s). Firstly, univariate analysis is conducted where the slope of each treatment arm is evaluated as different from the common slope of the other EBA confirmed arms. Thereafter, the treatment with the highest significant difference in TTP slope compared to the other arms in the univariate step, will be kept in the model. The second highest statistically significant treatment arm TTP slope will be added thereafter and statistically evaluated at a significance level of 5%. This will be continued for all arms. Arms where no difference in TTP slope can be shown will be grouped together to the same slope and the final model will predict the same EBA for these regimens. In the last step, IIV in the different TTP (and/or CFU) slopes are re-evaluated. It is important to note that EBA trials often are powered to only detected EBA and not to identify differences between arms. There might therefore not be sufficient power to detected small differences between treatments given the commonly used sample size for EBA trials.

#### 2.1.8 Early bactericidal activity reporting

All sections mentioned above should be included in an analysis report (European Medicines Agency, 2008; Food and Drug Administration, 2022). RMarkdown is a powerful tool to reproducibly compile reports on the graphical and numerical exploratory analysis in pdf or word format that are readily sharable with collaborators for review (Allaire et al., 2020; van Wijk et al., 2022). In addition to the sections presented above, reports should also include final parameter estimates from the model and typical and individual model-based predictions. Model-based typical and individual CFU or TTP predictions over time derived using individual Bayes estimates and converted to EBA_0-2_ (expressed as a difference in biomarker value between day 0 and day 2), EBA_0-7_, and EBA_0-14_ together with plots of predictions can be used to compare the activity of the treatments that were shown to have EBA, irrespective of the function applied to describe data.

### 2.2 Sample size to detect early bactericidal activity

Power is defined as the probability of rejecting the null hypothesis correctly (Eq. 13):

$$\begin{array}{c} Power=1-\beta \end{array}$$ (13)

where β is the probability of type II error (i.e., false negative). In EBA trials, the null hypothesis is most often defined as that the treatment or drug has no EBA.

Monte-Carlo Mapped Power (MCMP) method is a model-based approach to derive power curves for scenarios of interest (Vong et al., 2012). MCMP method was used in this work to calculate the power needed to detect EBA given different TTP slopes and variability in TTP slope. MCMP contains two parts; simulation and estimation, followed by power mapping (Vong et al., 2012). In the simulation and estimation step, MCMP simulates initially a large dataset. In this work, 2000 individuals were simulated with a full TTP-EBA model including an EBA efficacy for the two treatments as well as an EBA difference between the two treatments seen as two different TTP slopes and a difference between the slopes (Supplementary Table S2). In liquid culture experiments, the maximum incubation period is 42 days, therefore simulated TTP values greater than 42 days were censored at 42 days (1,008 h). This large simulated dataset was re-estimated with the full and reduced models. The reduced model was an TTP-EBA model with a TTP-slope fixed to zero (i.e., no EBA). Individual objective function values (iOFVs) from both the full and reduced models were used to calculate the difference in iOFV for each individual (∆iOFV). ∆iOFVs were taken to the power mapping step, where Monte Carlo sampling was performed, and the sum of ∆iOFVs was derived multiple times (10,000 times) for each sample size. Power at each sample size was derived based on the number of Monte Carlo sampling instances where the sum of ∆iOFV was greater than the critical χ^2^ value (for *p* < 0.05) divided by the total number of Monte Carlo sampling instances performed. Power was presented for up to 30 individuals in the trial scenarios. The goal in every analysis was to reach 80% power at a 5% significance level.

**FIGURE 2:**
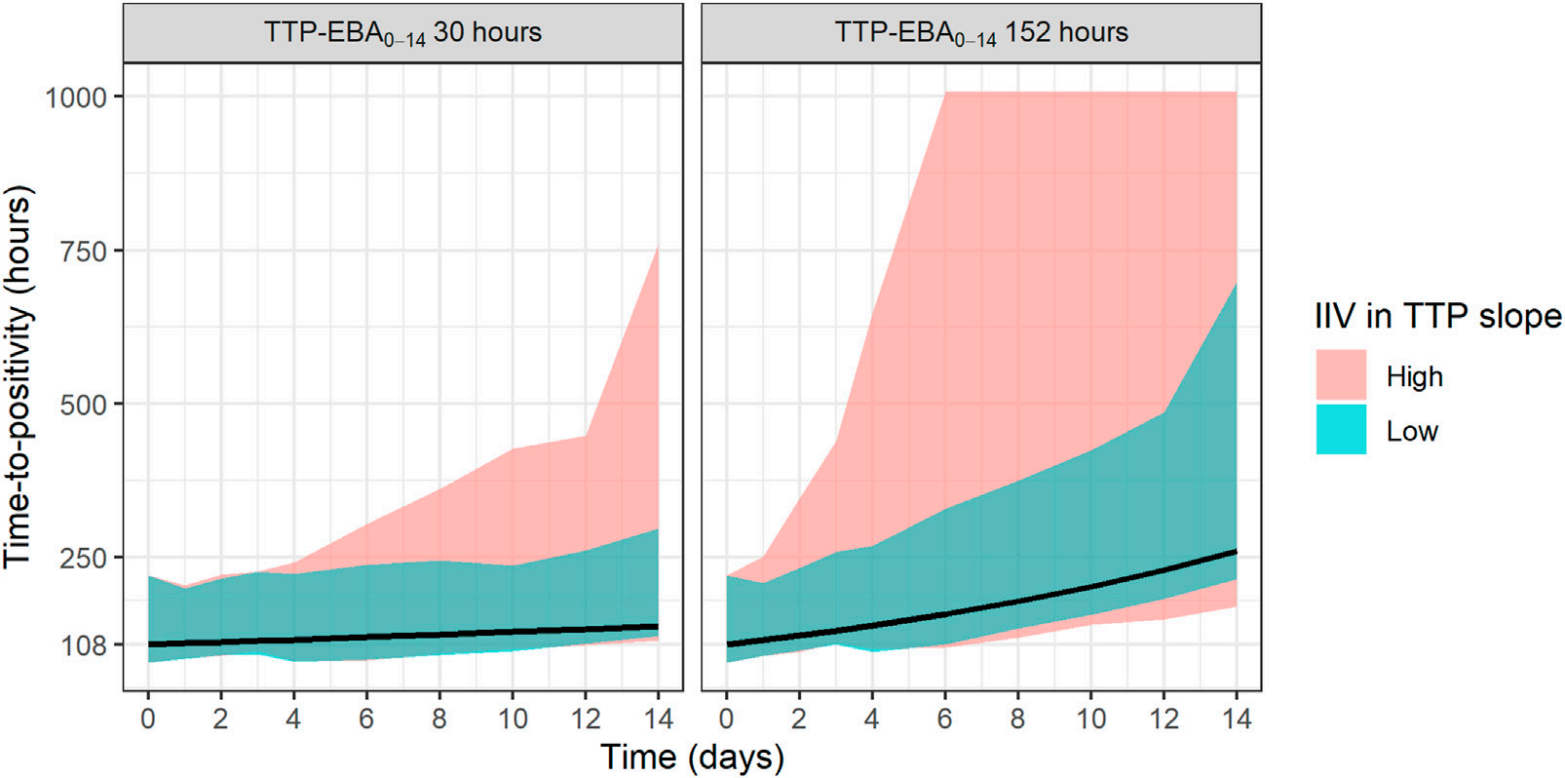
Visualization of typical profiles and variability in time-to-positivity (TTP) over time for high [104% coefficient of variation (CV)] and low (22% CV) inter-individual variability (IIV) in EBA. TTP-EBA_0-14_ is early bactericidal activity based on the difference in TTP between 0 and 14 days. Black lines represent the typical individual and shaded areas show the variability in predicted TTP derived from 30 randomly sampled individuals (95% prediction interval). The median baseline TTP was 1,008 h.

The sample sizes needed to detect EBA in relation to the influence of various TTP slopes and IIV in TTP slope (low and high IIV in TTP slope) was investigated. Explored IIV in TTP slopes were 22% coefficient of variation (CV) for low IIV in TTP slope, and 104% CV for high IIV in TTP slope. This was taken from the earlier quantified IIV in TTP slope from the two trials with meropenem presented in De Jager et al. (De Jager et al., 2022) and unpublished data (ClinicalTrials.gov Identifier: NCT04629378). TTP slope values ranged from 0.0017 h/day to 0.0628 h/day, corresponding to TTP-EBA_0-14_ of 3 h and 152 h, respectively. The different explored scenarios of TTP slope (TTP-EBA_0-14_) and IIV in TTP slope are presented in Supplementary Table S3. Visualization of typical profiles and variability in TTP over time for high (104%) and low (22%) IIV in TTP slope based on 30 simulated participants are shown in Figure 2.

### 2.3 Sample size to detect treatment effect difference

**FIGURE 3:**
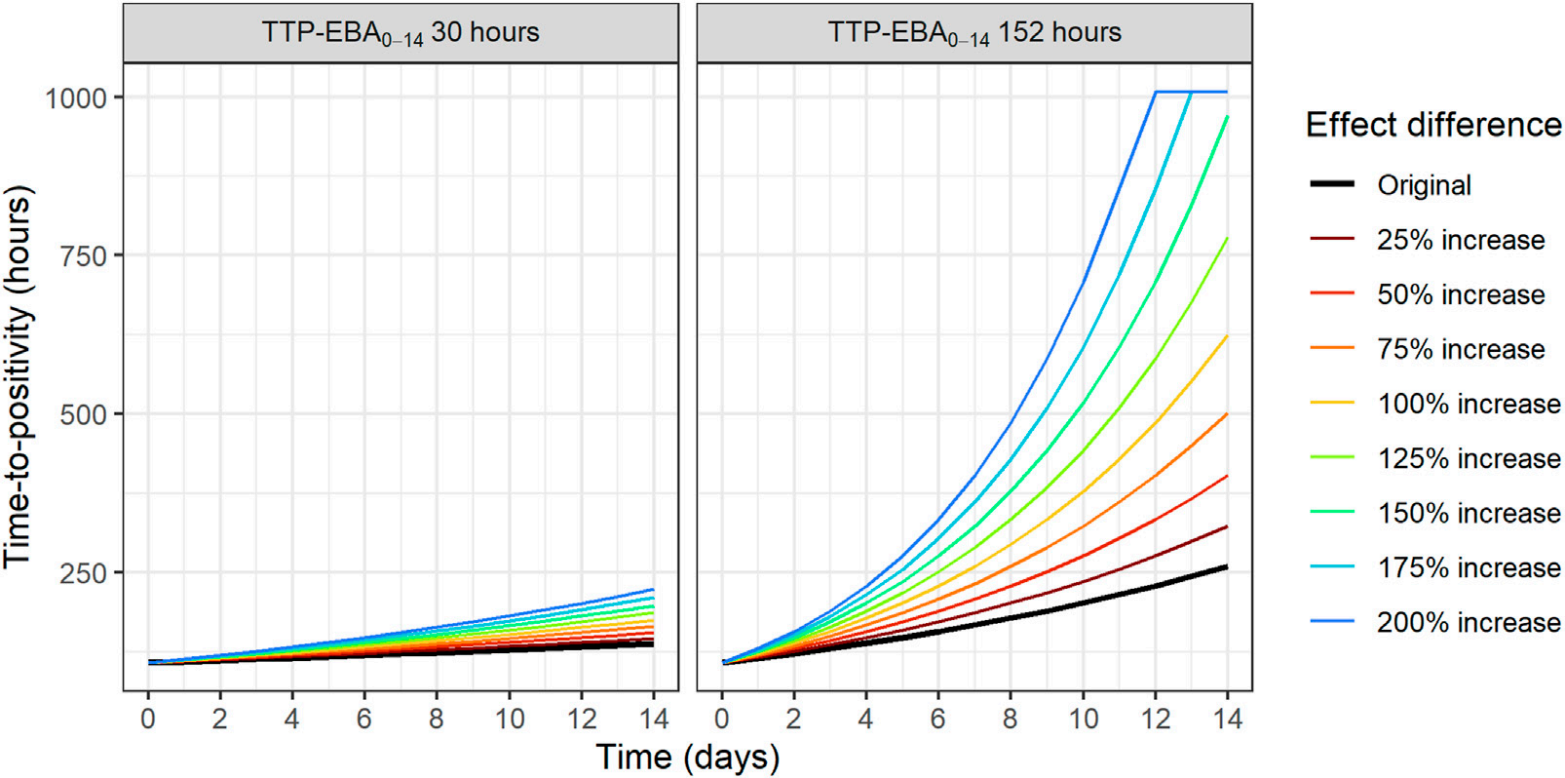
Visualization of typical profiles of time-to-positivity (TTP) over time for different effect difference values. Effect difference is expressed as a percentage increase in the TTP slope compared to the original value (red solid line). TTP values were right censored at 1,008 h (42 days).

To identify the sample sizes needed to detect a difference in EBA between two treatments, the null hypothesis was that there is no difference in EBA between the two treatments. In this work, power for different sample sizes were evaluated for scenarios with different EBA between two treatment groups. In addition, the influence of different IIV in TTP slope on the power for different sample sizes were explored. The MCMP approach described above was used. Effect difference was defined as a percentage increase in TTP slope between two treatments. Combinations of different TTP slopes, IIV in TTP slope and difference in TTP slope (from 25% to 200%) were investigated; TTP slope of 0.0628 h/day (TTP-EBA_0-14_ of 152 h) with low IIV in TTP slope; TTP slope of 0.0628 h/day (TTP-EBA_0-14_ of 152 h) with high IIV in TTP slope; TTP slope of 0.0174 h/day (TTP-EBA_0-14_ of 30 h) with low IIV in TTP slope; and TTP slope of 0.0174 h/day (TTP-EBA_0-14_ of 30 h) with high IIV in TTP slope. High IIV and low IIV in TTP slope were defined as 104% and 22%, respectively based on earlier reported variabilities in De Jager et al. (De Jager et al., 2022) and unpublished data (ClinicalTrials.gov Identifier: NCT04629378). Created scenarios are presented in Supplementary Table S4. Visualization of typical profiles of TTP over time for different effect differences are presented in Figure 3.

To characterize the influence of IIV in TTP slope on the power to detect EBA effect difference, TTP slope of 0.0628 h/day and 0.0174 h/day (TTP-EBA_0-14_ of 152 h and 30 h) were combined with different magnitudes of IIV in TTP slope. The same EBA effect difference of 50% was used in all scenarios and IIV in TTP slope spanned from high IIV (104%) to low IIV (10%). Explored scenarios are presented in Supplementary Table S5.

### 2.4 Software

Data handling and visualization were performed using R (v.4.0.3, R Foundation for Statistical Computing, Vienna, Austria) (R Core Team, 2020) through the RStudio interface (RStudio Team, 2022). Simulations were performed using non-linear mixed-effects modeling software NONMEM (v.7.5.0, Icon Development Solutions, Ellicott City, MD, United States) (Beal et al., 1989). MCMP were performed using PsN (v.5.3.0) (https://uupharmacometrics.github.io/PsN/).

## 3 Results

### 3.1 Standardized pharmacometric model-based early bactericidal activity analysis approach

In order to present each step of the standardized pharmacometric model-based analysis approach for Phase 2a EBA trials (Figure 1), each step was demonstrated using a simulated example based on a model resembling meropenem-containing treatments (Supplementary Table S1). The work is illustrated for TTP but is applicable to CFU as well.

#### 3.1.1 Exploratory data analysis

Before the modeling was started, data exploration to establish an analysis dataset from the raw data should be performed. Firstly, data summary tables were created based on the simulated data. The simulated number of participants and covariate information per treatment group and in total are presented in Supplementary Table S6. Counts of non-positive and censored data at different timepoints are presented in Supplementary Table S7.

**FIGURE 4:**
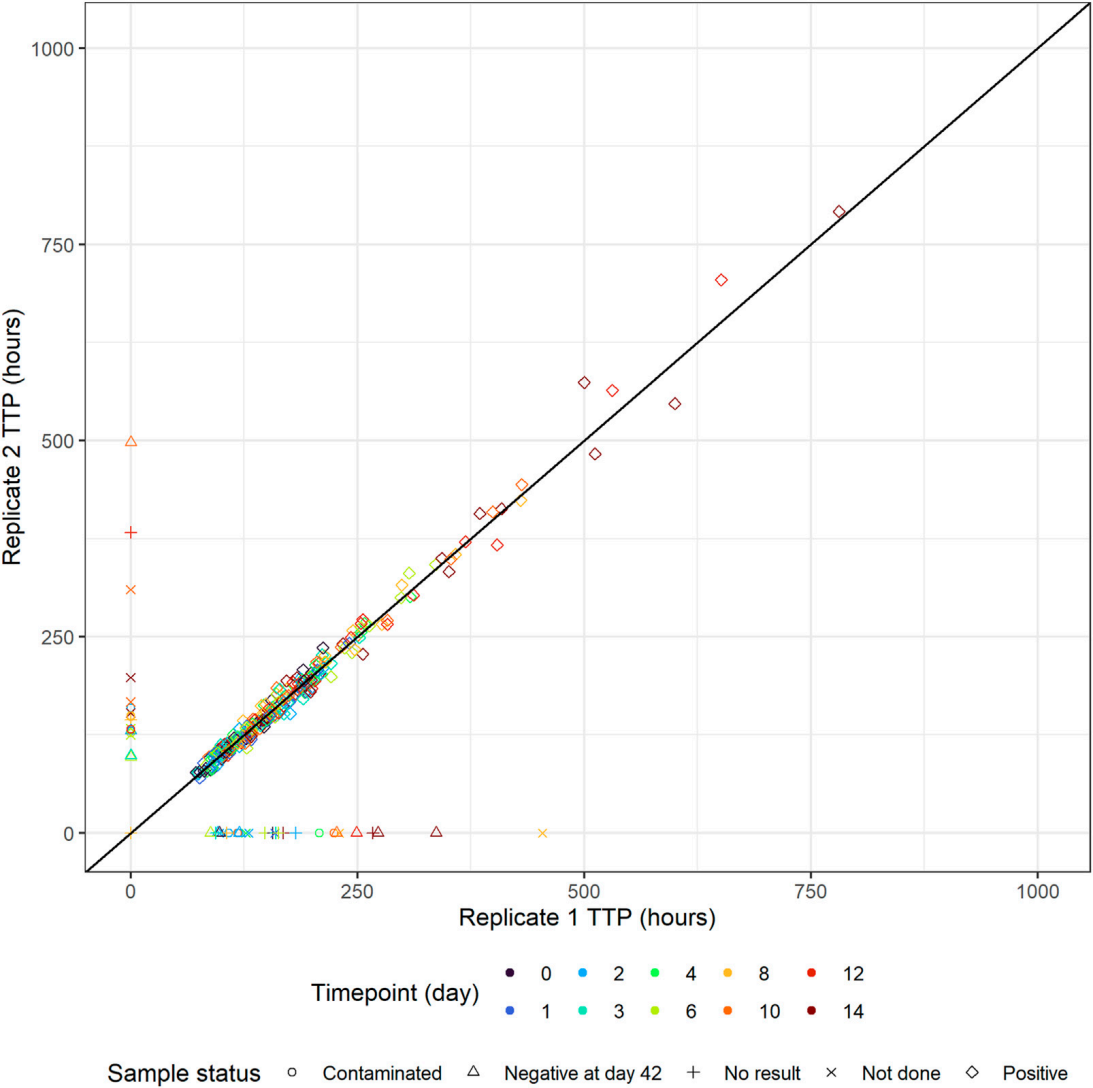
Individual time-to-positivity (TTP) replicate 1 *versus* individual TTP replicate 2. Observations are expected to be as close to the identity line as possible, and any clear deviations from the identity line should be investigated. Non-positive values are plotted in the figure axes and their symbol represents the corresponding sample status. For example, if replicate 1 is non-positive, the symbol with the corresponding replicate 2 value is plotted on the *y*-axis and *vice versa*.

**FIGURE 5:**
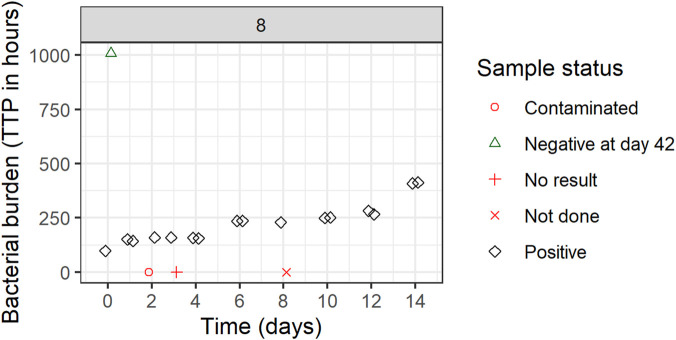
Individual biomarker over time plot. Both replicates 1 and 2 are plotted which can seem to overlap because of very comparable values. One participant was selected to visualize the status of each sample. If a replicate value was non-positive, it was plotted in red (missing) or green (negative) on the graph axes with the shape informing on the reason for its non-positive sample status. These symbols are plotted next to each other to facilitate interpretation but belong to the same time point. Plots are based on the simulated data to visualize the standardized pharmacometric model-based EBA analysis approach. Plots of individual biomarker over time for all individuals are available in Supplementary Figure S1.

Replicate-versus-replicate of the raw data was plotted and any clear deviations from the identity line were further investigated (Figure 4). Additionally, biomarker *versus* time of TTP was plotted to facilitate data comparison between the replicates and over time. An example of an individual plot is shown in Figure 5 (for subject 8 only). Replicates are plotted next to each other to facilitate comparison, while individual plots for all subjects are available in Supplementary Figure S1.

All TTP samples with the status of contaminated, not done, or no result were excluded from the model-based analysis. After discussing the results with clinicians and microbiologists, only samples with scientifically plausible TTP values were kept, and any clear deviations that have potentially arisen due to the experiment errors, unrecorded contamination, or other reasons were removed from the analysis. In Figure 5 for example, the negative sample at time zero is a clear outlier and was removed from the analysis after consensus was reached with clinical, microbiology, and analysis teams. All removed and omitted data points should be summarized in the final report.

After the final analysis dataset was established, additional plots were created showing the relationship between the baseline biomarker and covariates, and biomarker over time and covariates. Supplementary Figure S2 shows the TTP baseline plots *versus* covariates present in the simulated dataset; sex, age and cavity extent. In addition, plots of TTP over time *versus* cavity extent, sex, age, PK drug exposure (AUC), and treatment. These plots should be used to inform the modeling workflow by informing about the potential function describing the change in the biomarker over time as well as about potential parameter-covariate relationships.

#### 3.1.2 Base model building

After the analysis dataset is established, a base model should be developed. In this simulation based EBA example, the mono-exponential function described the data well. IIV was supported on baseline and TTP slope. The proportional error model was used with both common and replicate-specific error terms for each replicate. Goodness-of-fit (GOF) plots evaluating the model fit are presented in Supplementary Figures S3A-E. Visual predictive check (VPC) plots stratified per treatment arm are presented in Supplementary Figure S4, column a. Based on GOFs and VPCs stratified on treatment, a satisfactory fit was achieved using a mono-exponential function with IIV on baseline and slope, and the model was carried forward to the covariate modeling.

#### 3.1.3 Covariate model building

In the next step, covariate model building is performed. Age, sex, and cavity extent were tested on both the TTP baseline (intercept) and the TTP slope. Only one covariate was found to be statistically significant after the forward inclusion and backwards deletion step in the SCM. Decrease in TTP baseline values was associated worsening cavity extent states, and a separate TTP baseline was estimated for each category (*p* < 0.01). The highest TTP baseline was predicted when no cavities were present. VPCs stratified on cavity are presented in Supplementary Figure S5.

#### 3.1.4 Early bactericidal activity detection

After the covariate model is built, EBA detection can be performed. Here, in this example, both treatment arms A and B were shown to have EBA at a 5% significance level.

#### 3.1.5 Pharmacokinetic-pharmacodynamic modeling

All treatment arms that were shown to have EBA, were further taken to build the PK-PD model, where a relationship between AUC of meropenem and TTP slope was assessed. Linear, hockey-stick, power and exponential covariate-parameter relationships were evaluated on natural logarithm scale. Out of all assessed parameterizations, the hockey-stick function had the biggest change in objective function value (∆OFV) (−49.866) but as one of the parameters had very high uncertainty, a linear parameterization with second biggest ∆OFV (−45.463) was selected, which resulted in an exponential relationship on normal scale. Additionally, after meropenem AUC was incorporated as a covariate on the TTP slope, IIV in TTP slope decreased from 49.7% to 20.5%, explaining the PK related variability between subjects. VPCs stratified on meropenem AUC are presented in Supplementary Figure S6.

#### 3.1.6 Early bactericidal activity comparison

After PK-PD modeling, a treatment comparison can be performed. In this work, treatment comparison between simulated Arm A and Arm B was performed. Treatments were shown to have statistically significantly different EBAs from each other at a 5% significance level. Arm B was shown to have 3.7 times higher EBA compared to Arm A in this simulated example.

#### 3.1.7 Early bactericidal activity reporting

The final TTP-EBA model described the data well and the final parameters are shown in Supplementary Table S8. The final TTP-EBA model consisted of a mono-exponential model with IIV in baseline (intercept) and TTP slope. Covariate relationships were cavity extent on TTP baseline and AUC meropenem on TTP slope. A proportional error model with both common and replicate-specific error terms for each replicate was included in the final model. GOF plots are presented in Supplementary Figure S3F-J, stratified VPCs per treatment arm and prediction-corrected VPC are available in Supplementary Figure S7 and Supplementary Figure S4 column b.

**FIGURE 6:**
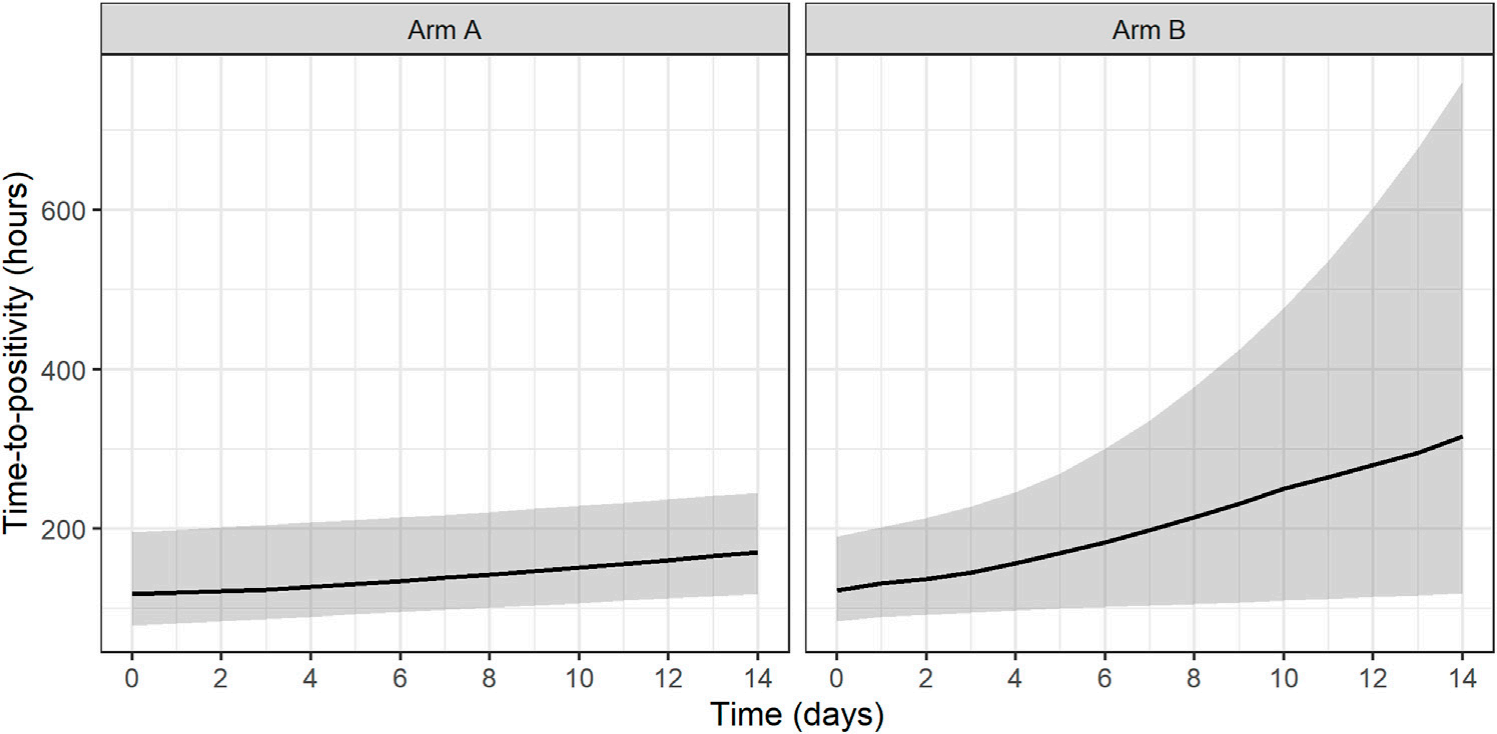
Prediction of individual time-to-positivity (TTP) over time based on Bayes estimates of the final model. Lines represent the predicted median with a shaded area corresponding to the 95% prediction interval. Arm A resembling meropenem in 2 g meropenem thrice daily with 500 mg amoxicillin and 125 mg clavulanate thrice daily on days 1–14 (De Jager et al., 2022). Arm B resembling meropenem in 6 g meropenem once daily with 2 × 1000 mg amoxicillin and 62.5 mg clavulanate plus 400 mg bedaquiline once daily on days 1–14 (unpublished data, ClinicalTrials.gov Identifier: NCT04629378).

Both treatments based on simulated data for Arm A and Arm B were shown to have statistically significant EBA (*p* < 0.05). In addition, simulated Arm B was shown to have statistically significantly higher TTP slope than simulated Arm A (3.7 times higher TTP slope). Predicted typical and individual model-based EBA for 0–2, 0–7, and 0–14 days were derived. Prediction of individual TTP over time using Bayes estimates of the final model is shown in Figure 6. Predicted typical and individual model-based EBA for 0–2, 0–7, and 0–14 days are presented in Supplementary Tables S9, 10.

**FIGURE 7:**
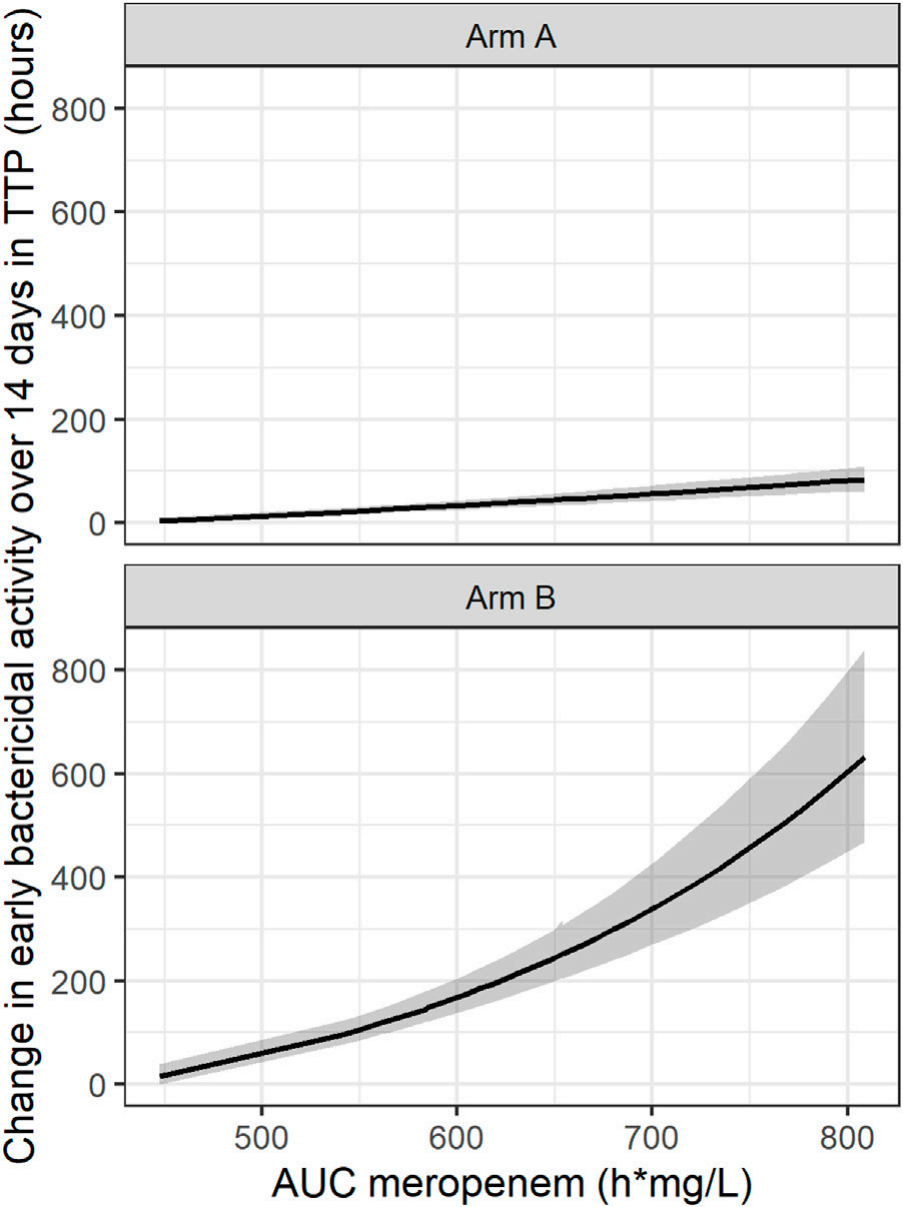
Established pharmacokinetic-pharmacodynamic (PK-PD) relationship between area under the curve (AUC) of meropenem and early bactericidal activity (EBA) based on time-to-positivity of 0–14 days (TTP-EBA_0-14_) for the typical individual with one or several cavities <4 cm. The black line represents the median and the shaded area represents the 95% confidence interval. 95% confidence interval is derived from Sampling Importance Resampling (SIR) method followed by Stochastic Simulation and Estimation (SSE) step of 1,000 samples. Arm A resembling meropenem in 2 g meropenem thrice daily with 500 mg amoxicillin and 125 mg clavulanate thrice daily on days 1–14 (De Jager et al., 2022). Arm B resembling meropenem in 6 g meropenem once daily with 2 × 1000 mg amoxicillin and 62.5 mg clavulanate plus 400 mg bedaquiline once daily on days 1–14 (unpublished data, ClinicalTrials.gov Identifier: NCT04629378).

In addition, an exponential PK-PD relationship between the meropenem AUC and TTP slope and thereby TTP-EBA_0-14_ was established. In this simulated example, TTP increased by 0.44 h per each 1 h mg/L meropenem AUC_0-inf_ on the natural logarithm scale (Figure 7).

### 3.2 Sample size to detect early bactericidal activity

To investigate the sample size needed to detect EBA, eight different TTP slopes (0.0017 h/day to 0.0628 h/day) corresponding to TTP-EBA_0-14_ values ranging from 3 h to 152 h were combined with low and high IIV in TTP slope (22% and 104%, respectively). In total, 16 scenarios were explored (Supplementary Table S3). Simulated typical profiles and corresponding variability based on 30 individuals (expressed as a 95% prediction interval) are visualized for both low and high variability in TTP slope (22% and 104%) and TTP-EBA_0-14_ values of 30 h (slope 0.0174 h/day) and 152 h (slope 0.0627 h/day) (Figure 2).

**FIGURE 8:**
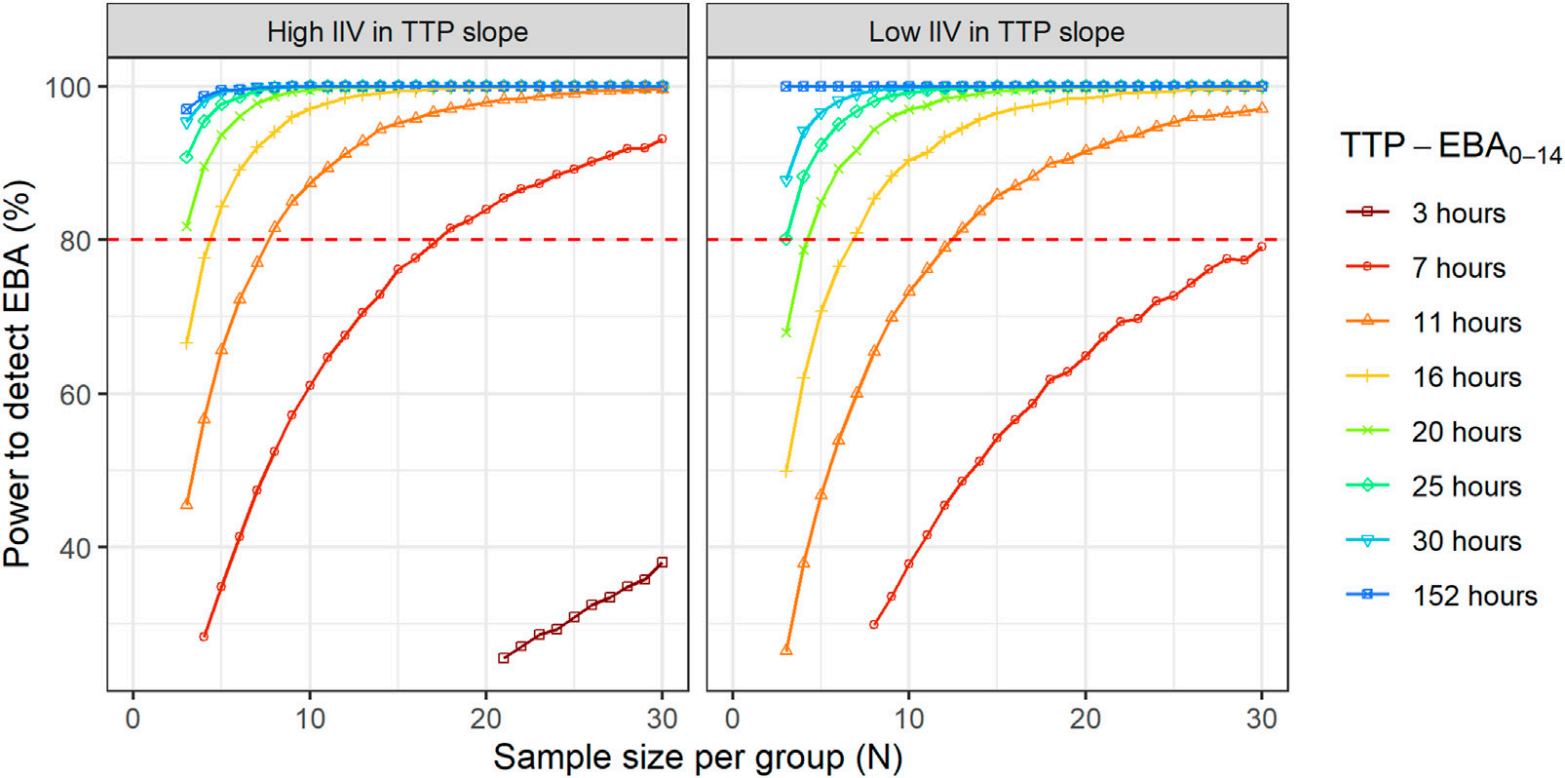
The impact of change in time-to-positivity between days 0 and 14 (TTP-EBA_0-14_) on sample size per arm and power to detect early bactericidal activity (EBA). Low and high inter-individual variability (IIV) in TTP slope are represented by 22% and 104% coefficient of variation (CV). The red dashed line represents 80% power.

Sample sizes per arm needed to detect EBA with 80% power at a 5% significance level are presented in Table 1 and power curves are presented in Figure 8 for the difference scenarios. TTP-EBA_0-14_ had to be equal to or greater than 11 h, irrespective of IIV in TTP slope, to achieve 80% power at a 5% significance level with 13 participants enrolled per treatment arm. An increase in sample size, to 18 participants per arm, resulted in a study having 80% power to detect EBA when TTP-EBA_0-14_ was 7 h and high IIV in TTP slope was present (104%). For a scenario with low IIV in TTP slope (22%) 31 participants/arm were required to reach the same power. If the TTP-EBA_0-14_ was 3 h, 97 and 180 participants/arm for high IIV and low IIV, respectively, were required to achieve 80% power to detect TTP-EBA_0-14_.

**TABLE 1:** Sample size per group needed to detect early bactericidal activity (EBA) with 80% power at a 5% significance level for various changes in time-to-positivity between days 0 and 14 (TTP-EBA_0-14_) and inter-individual variability (IIV) in TTP slope. Power calculations were performed using a mono-exponential model with various TTP-EBA_0-14_ values for both low and high IIV in TTP slope. For each scenario, power calculations included a minimum of 3 patients.

TTP-EBA_0-14_ ^a^	N per treatment group	
	Low IIV in TTP slope^b^	High IIV in TTP slope^c^
152 h^d^	3	3
30 h^e^	3	3
25 h	3	3
20 h	5	3
16 h	7	5
11 h	13	8
7 h	31	18
3 h	180	97

^a^ TTP-EBA_0-14_: typical change in TTP between day 0 and day 14 (expressed in hours).

^b^ Low IIV in TTP slope was 22% coefficient of variation (unpublished data, ClinicalTrials.gov Identifier: NCT04629378).

^c^ High IIV in TTP slope was 104% coefficient of variation (De Jager et al., 2022).

^d^ TTP-EBA_0-14_ corresponding to treatment composed of 6 g meropenem once daily with 2 × 1,000 mg amoxicillin and 62.5 mg clavulanate plus 400 mg bedaquiline once daily on days 1–14 (unpublished data, ClinicalTrials.gov Identifier: NCT04629378).

^e^ TTP-EBA_0-14_ corresponding to treatment composed of 2 g meropenem thrice daily with 500 mg amoxicillin and 125 mg clavulanate thrice daily on days 1–14 (De Jager et al., 2022).

EBA: early bactericidal activity.

IIV: inter-individual variability expressed on coefficient of variation scale.

**FIGURE 9:**
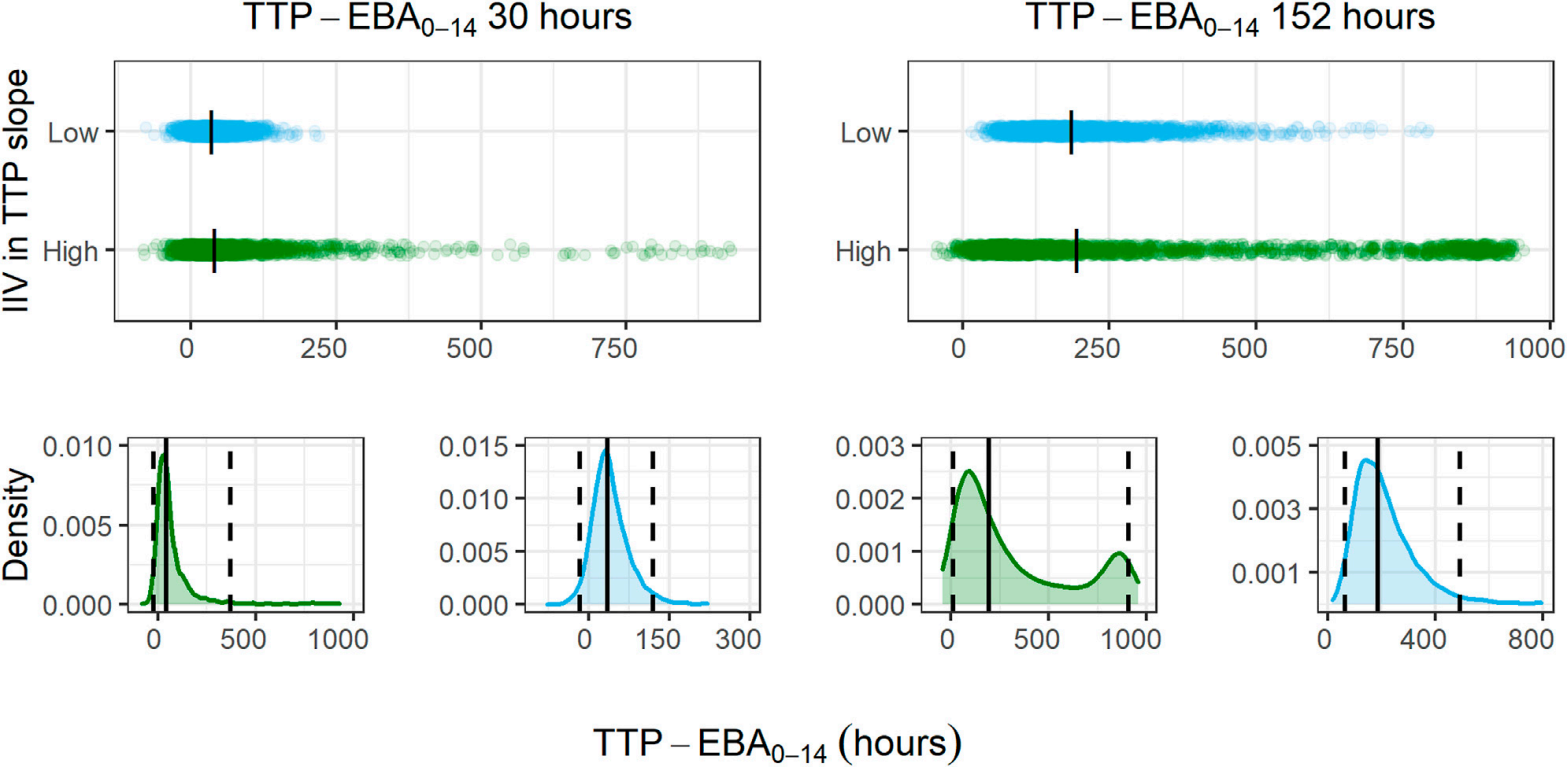
Distribution of individual simulated change in time-to-positivity between days 0 and 14 (TTP-EBA_0-14_) values for two TTP-EBA_0-14_ values; 30 h and 152 h, for different magnitudes of inter-individual variability (IIV) in TTP slope. The blue color is low IIV in EBA (22% coefficient of variation [CV]), and the green color is high IIV in TTP slope (104%). To visualize the spread of values, 2000 individuals per scenario of IIV in TTP slope and TTP-EBA_0-14_ were simulated, and the difference between TTP on day 0 and day 14 was calculated. The top panel of the plot shows the spread of individual TTP-EBA_0-14_ values over the TTP-EBA_0-14_ space. Black dashes represent individual medians. The bottom panels show density plots for the individual TTP-EBA_0-14_ values. A solid black line represents the median, and dashed lines represent the 2.5th and 97.5^th^ percentiles of IIVs. The second peak in TTP-EBA_0-14_ of 152 h and high IIV in TTP slope was related to TTP censoring at 1,008 h.

For treatments showing TTP-EBA_0-14_ of 30 h or less (typical prediction), the power to detect the EBA given the same sample size, was higher when IIV was larger. This trend was present for even lower TTP-EBA_0-14_ values (Figure 8). The posthoc step of the Bayes estimates of the IIV showed that the mean of the individual etas was skewed towards large positive values of the TTP slope (Figure 9). Due to this, when low EBA effect (TTP-EBA_0-14_ of 30 h or less) was combined with high IIV in TTP slope, the probability of having a significant EBA was higher compared to a scenario where low EBA was combined with low IIV. Opposite effect was observed with high EBA (TTP-EBA_0-14_ of 152 h), as the distribution of individual TTP-EBA_0-14_ values was narrower (Figure 9) and very few extremely low TTP slope values were present.

### 3.3 Sample size to detect treatment effect difference

**FIGURE 10:**
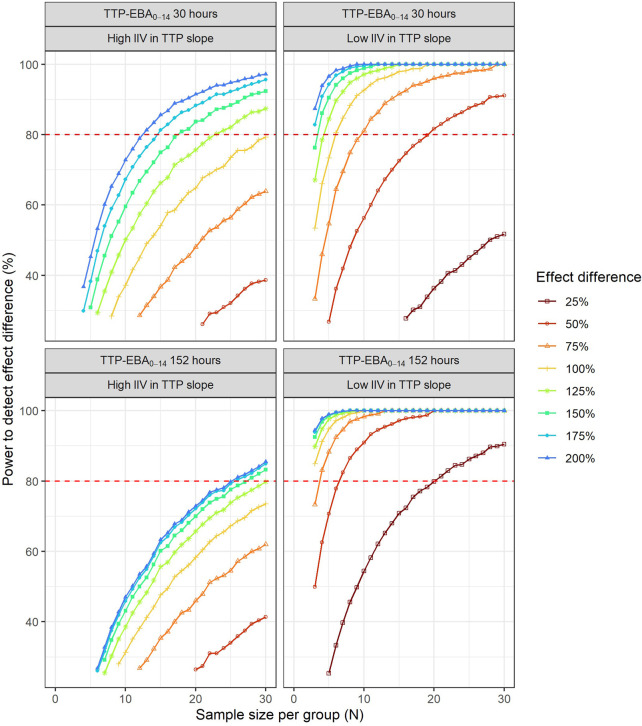
The impact of effect difference on the sample size and power to detect a difference between two treatment groups. Low and high inter-individual variability (IIV) in early bactericidal activity (EBA) are represented by 22% and 104% coefficient of variation, respectively. The red dashed line represents 80% power.

To characterize the sample size needed to detect an effect difference between two treatment groups, several different scenarios with various combinations of different TTP slope (TTP-EBA_0-14_), IIV in TTP slope and effect difference values were explored (Supplementary Table S4). Simulated typical profiles for the different effect differences are presented in Figure 3. Power curves and a summary table presenting the number of participants per group required to achieve 80% power are presented in Figure 10 and Table 2.

**TABLE 2:** Sample size per group in an early bactericidal activity (EBA) clinical trial needed to detect an effect difference between two treatment groups with 80% power at a 5% significance level. Power calculations were performed using a mono-exponential time-to-positivity (TTP) model for different change in TTP between days 0 and 14 (TTP-EBA_0-14_), inter-individual variability (IIV) in TTP slope, and increased effect difference values. For each scenario, power calculations included a minimum of 3 patients.

Effect difference between two treatments^a^	N per treatment group			
	TTP-EBA0-14 of 30 hours^b^		TTP-EBA0-14 of 152 hours^c^	
	Low IIV in TTP slope^d^	High IIV in TTP slope^e^	Low IIV in TTP slope^d^	High IIV in TTP slope^e^
+25%	64	>125	21	>125
+50%	20	90	7	87
+75%	10	48	3	50
+100%	6	31	3	37
+125%	5	23	3	31
+150%	4	18	3	28
+175%	3	15	3	26
+200%	3	13	3	26

^a^ Increase in TTP-EBA_0-14_ between two drugs.

^b^ TTP-EBA_0-14_ corresponding to treatment composed of 2 g meropenem thrice daily with 500 mg amoxicillin and 125 mg clavulanate thrice daily on days 1–14 (De Jager et al., 2022).

^c^ TTP-EBA_0-14_ corresponding to treatment composed of 6 g meropenem once daily with 2 × 1,000 mg amoxicillin and 62.5 mg clavulanate plus 400 mg bedaquiline once daily on days 1–14 (unpublished data, ClinicalTrials.gov Identifier: NCT04629378).

^d^ Low IIV in TTP slope was 22% coefficient of variation (unpublished data, ClinicalTrials.gov Identifier: NCT04629378).

^e^ High IIV in TTP slope was 104% coefficient of variation (De Jager et al., 2022).

EBA: early bactericidal activity.

IIV: inter-individual variability expressed on coefficient of variation scale.

For low TTP-EBA_0-14_ (30 h), high IIV in TTP slope and an increased effect difference between the two arms of at least 175%, 15 participants/arm were sufficient to detect a difference between the two treatment groups with 80% power at a 5% significance level (Table 2; Figure 10). At 30 participants/arm, the power was ≥80% to detect an increased effect difference between the two arms of at least 125%. To detect smaller increased effect differences (<100%), more participants were required. For an increased effect difference of 25% at 80% power, more than 125 participants/arm were needed to detect an effect difference between the two arms.

For high TTP-EBA_0-14_ (152 h) and high IIV in TTP slope, 80% power was only reached when the increased effect difference was at least 150% between the two arms and with 28 participants/arm. None of the explored scenarios reached 80% power with 15 participants/arm or less. To detect 125% increased effect difference, 31 participants/arm were required. For even smaller increased effect differences of 100%, 75%, and 25%, sample sizes of 50, 87 and >125 participants, respectively were required at 80% power and for high TTP-EBA_0-14_ (152 h) and high IIV in TTP slope (Table 2).

At low TTP-EBA_0-14_ (30 h) and low IIV in TTP slope, an increased effect difference of 75% or greater was detected with 15 participants/arm for at least 80% power, while 20 participants/arm were required to detect an increased effect difference of 50%. Similar, 64 participants/arm were required to detect an increased 25% effect difference (Table 2; Figure 10).

For all effect differences, the highest power was seen for scenarios with low IIV in TTP slope and high TTP-EBA_0-14_ (152 h). Less than 15 participants/arm were required for increased effect differences of ≤50%. To detect a 25% increased effect difference between the two arms, 21 participants/arm were sufficient to achieve 80% power at a 5% significance level. For other scenarios, more participants were needed to differentiate between the treatments at this effect difference level (Table 2; Figure 10).

**FIGURE 11:**
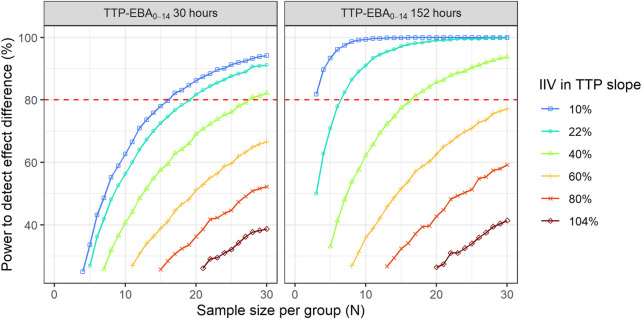
The impact of inter-individual variability (IIV) in early bactericidal activity (EBA) on the sample size and power to detect a difference between two treatment groups. The effect difference between the two treatment groups was set to 50%. The red dashed line represents 80% power.

To explore IIV in TTP slope on the power to detect an effect difference between the two arms, a scenario with a 50% increase in effect difference was used. IIV in TTP slope in the simulations ranged from very low to very high IIV in TTP slope, 10% CV to 104% CV (Supplementary Table S5). Power curves and corresponding numbers of participants/arm needed to achieve 80% power are presented in Figure 11 and Table 3.

**TABLE 3:** Sample size per group in an early bactericidal activity (EBA) clinical trial needed to detect an effect difference between two treatment groups with 80% power at a 5% significance level under different inter-individual variability (IIV) in time-to-positivity (TTP) slope. Power calculations were performed using a mono-exponential model with an effect difference between two treatment groups of 50%, various changes in TTP between days 0 and 14 (TTP-EBA_0-14_), and inter-individual variability (IIV) in TTP slope. For each scenario, power calculations included a minimum of 3 patients.

IIV in TTP slope (%)	N per treatment group	
	TTP-EBA_0-14_ of 30 h^a^	TTP-EBA_0-14_ of 152 h^b^
10	17	3
22	20	7
40	28	17
60	44	33
80	63	54
104	90	87

^a^TTP-EBA_0-14_ corresponding to treatment composed of 2 g meropenem thrice daily with 500 mg amoxicillin and 125 mg clavulanate thrice daily on days 1–14 (De Jager et al., 2022).

^b^TTP-EBA_0-14_ corresponding to treatment composed of 6 g meropenem once daily with 2 × 1,000 mg amoxicillin and 62.5 mg clavulanate plus 400 mg bedaquiline once daily on days 1–14 (unpublished data, ClinicalTrials.gov Identifier: NCT04629378).

EBA: early bactericidal activity.

CV: coefficient of variation.

IIV: inter-individual variability expressed on coefficient of variation scale.

When TTP-EBA_0-14_ was low (30 h), 90 participants/arm were required to achieve 80% power to detect 50% increase in effect difference between the two treatment groups with IIV in TTP slope of 104%. With a decrease in IIV in TTP slope to 40%, the sample size decreased to 28 participants/arm (Table 3). When IIV in TTP slope was 10% and 50% increase in effect difference, 17 participants/arm were required to reach 80% power for a low TTP-EBA_0-14_ (30 h).

For cases with high TTP-EBA_0-14_ (152 h) and IIV in TTP slope of 104%, 87 participants/arm were required to achieve 80% power to detect an increase of 50% effect difference between the two treatment groups. Decreasing the IIV in TTP slope to 40% resulted in a decreased sample size of 17 participants/arm. Less than seven and three participants/arm were required to achieve a power of 80% when IIV in TTP slope was 22% or 10%, respectively (Table 3; Figure 11).

## 4 Discussion

This paper presents a standardized pharmacometric model-based EBA analysis approach employing the expertise of microbiologists, clinicians and pharmacometricians. The presented approach is composed of seven pivotal steps, which allows for both model-based pharmacometric EBA detection and treatment comparison in Phase 2a for TB drug development, and is identical for evaluation of monotherapies as well as combination regimen. Using the standardized approach, the treatment/drug effect is evaluated through the TTP slope estimation together with uncertainty. It allows for a rational estimation of EBA, as both typical and individual predictions can be derived to perform treatment comparisons, describing the PD response on both the population and individual levels. These model-based predictions can be further used to make the decision for the treatments to be tested in the following phases supporting TB drug development.

The herein presented pharmacometric model-based EBA analysis approach has many advantages over traditional analysis approaches. It provides a robust way to account for covariates that might be influential on TTP slope, and explain variability in EBA. Covariate analysis can be turned into an automated process, where prespecified parameter-covariate relationships are tested, and a higher *p*-value can be set for a backward deletion step of covariates to protect from inflating type 1 error (Lindbom et al., 2005). As IIV reflects the variability on the population level, the application of covariate analysis which can reduce and explain variability in efficacy, i.e., TTP slope is therefore central for detecting treatment arm differences in EBA trial as the power will increase compared to not accounting for IIV. In this example, introducing drug exposure (meropenem AUC) as a covariate on TTP slope decreased the IIV in TTP slope by 29%. Using the standardized approach presented here, all factors contributing to the power to detect EBA and treatment differences between treatment arms can rationally be controlled for. As variability plays a pivotal role in EBA detection and treatment comparison, PK-PD model building is a critical step in the standardized pharmacometric model-based EBA analysis approach, as variability in PK translates into variability in PD, and incorporation of PK information in the model can decrease IIV in efficacy. Population PK models can be used to derive secondary PK parameters such as AUC, C_max_ and C_min_ to drive the PK-PD relationship as an alternative to use a PK-PD model linked to a population PK model. As such, the incorporation of secondary PK parameters into the EBA model may increase the power to detect EBA, and to find a difference between treatment groups by decreasing the variability in PD. Additionally, information about factors influencing variation in EBA biomarkers could be employed for individualized treatment, if deemed beneficial. Another advantage of PK-PD modeling over traditional EBA analysis is that half maximal effective concentration (EC_50_) can be estimated with a smaller sample size. In the traditional EBA modeling, dose would be driving the effect if several different dose levels are given, thus, variability in PK will be attributed to variability in EBA and more participants would be required for estimating EBA. Further, in a design with only one dose level, exposure cannot be attributed for in a traditional EBA analysis whereas in the pharmacometric model-based EBA analysis, between patient variability in drug exposure can still be accounted for through a PK summary indice which reduces the IIV in TTP slope and thereby leads to smaller sample sizes compared without accounting for between patient variability in drug exposure.

The presented workflow is built on similar techniques for model development and evaluation as presented in the FDA and EMA guidelines for analysis and reporting (European Medicines Agency, 2008; Food and Drug Administration, 2022). Koele et al. summarize the different historical analysis methods presented in the literature for analyzing EBA trials and concluded that a standardized analysis method, accounting for different levels of variability in the data, could aid the generalization of study results and facilitate comparison between drugs or treatments (Koele et al., 2023).

The presented standardized approach can be easily coupled with other modeling approaches, like the multistate tuberculosis pharmacometric (MTP) model. In the MTP model, data is analyzed using a semi-mechanistic approach describing different bacterial subpopulations (Clewe et al., 2016). This approach determined the contribution of clofazimine to TB treatment and its effect on persisters (Faraj et al., 2020), while it showed no EBA effect when analyzed using a hierarchical Bayesian non-linear mixed effects regression model (Diacon et al., 2015). Incorporation of semi-mechanistic features would help establish EBA when empirical methods are not able to as for the situation for drugs with predominant persister efficacy, and this would further improve drug development.

While the application of the standardized approach was exemplified using TTP, the same approach can be used for CFU as well. In addition, this standardized approach is not limited to CFU or TTP biomarkers and can be easily applied to others. Many novel sputum-based biomarkers have been developed in recent years and are currently in various stages of clinical validation and regulatory approval, and may provide a near real-time quantification of mycobacterial health and/or load, like the molecular bacterial load assay (MBLA) (Sabiiti et al., 2020), RNA synthesis ratio (RS ratio) (Walter et al., 2021), and the lipoarabinomannan enzyme-linked immunosorbent assay (LAM-ELISA) (Jones et al., 2022). Therefore, the approach can be extended to new biomarkers as most of the steps would remain the same, and only the structural model might be different. In addition, novel and more informative biomarkers that better captures efficacy on persistent bacteria will make an empirical model approach more informative.

In addition to providing a structured approach to EBA trial analysis, the models developed using this approach can be further used in other clinical trial simulations and model-based sample size determinations. To exemplify this, models used to visualize the modeling workflow were used in MCMP to characterize the number of participants needed to detect EBA and to detect a difference between two treatment groups with a significance level of 5% and a target power of 80%. Using the presented approach, even extremely low EBA (TTP-EBA_0-14_ of 11 h) 13 and 8 participants was sufficient to reach 80% power for low IIV in TTP slope (22%) and high IIV in TTP slope (104%) scenarios (Figure 8), and less participants were required for treatments with stronger EBA (Table 1).

Sample sizes to identify a treatment difference between two groups were characterized in addition to detecting EBA. Here, it was observed that for drugs with low IIV in TTP slope, irrespective of TTP-EBA_0-14_ value, a sample size of 15 participants/arm were sufficient to detect a difference in EBA with at least 80% power at a 5% significance level when the effect difference is at least 75% for TTP-EBA_0-14_ of 30 h or 50% for TTP-EBA_0-14_ of 152 h (Table 2; Figure 10). For scenarios with high IIV in TTP slope, larger sample sizes were required to ensure the same power, as high IIV dilutes the intrinsic difference between the two treatment arms (Table 2; Figure 10). This is seen in the results presented in Figure 11, where the sample size needed to achieve 80% power decreased with decreasing IIV in TTP slope, indicating the importance of decreasing IIV in TTP slope to increase the power where determination of an effect difference is sought after. These results are in accordance with other investigations, where the difference between arms was detected in CFU but not in TTP, as IIV was higher in TTP compared to CFU (104% CV *versus* 48% CV) (De Jager et al., 2022).

In a model-based analysis, preclinical data can be integrated to inform about the expected EBA, which can be used to identify the sample size needed to achieve the desired power. As shown in this study, the standard set-up with 15 participants per arm might not be suitable for all treatments and adjustments might be needed. While a set-up with 15 participants is enough to detect even low TTP-EBA_0-14_ of 11 h, the trial might be underpowered to detect a difference between two treatment groups. Due to this potential variability should be considered when designing the trial. One way to decrease IIV is to collect PK information in EBA studies and include this in the EBA analysis in order to increase the power and keep the sample sizes on the lower end. If no PK information is included in the EBA analysis, higher sample size should be enrolled into EBA trials.

In this work, an unexpected relationship between IIV in TTP slope and power to detect and/or compare the EBA of treatments was found. The scenario with less spread out individual TTP slope values (low IIV in TTP slope) resulted in a lower probability of overlapping confidence intervals between two arms, compared to high IIV in TTP slope. This subsequently led to a higher power to detect an effect difference when IIV in TTP slope was low, and the TTP slope itself had no impact here. While these results are in accordance with results from EBA detection of TTP-EBA_0-14_ 152 h (TTP slope 0.0627 h/day), an opposite effect was observed for low TTP slope values (0.0174 h/day and lower). Here, high IIV in TTP slope resulted in smaller sample sizes compared to scenarios with low IIV in TTP slope, as etas of IIV in TTP slope were skewed towards large positive values of the TTP slope.

In conclusion, a robust standardized pharmacometric model-based EBA analysis approach established in close collaboration between microbiologists, clinicians, and pharmacometricians was presented. The work illustrates the importance of accounting for covariates and drug exposure in EBA analysis in order to increase the power of detecting EBA for a single treatment arm as well as differences in EBA between treatments arms in Phase 2a trials of TB drug development.

## Data Availability

The original contributions presented in the study are included in the article/Supplementary Material, further inquiries can be directed to the corresponding author.
