# Supplementary material for: Standards for model-based early bactericidal activity analysis and sample size determination in tuberculosis drug development
Source: Front Pharmacol. 2023 Apr 13;14:1150243. doi: 10.3389/fphar.2023.1150243 (PMC10133723; doi:10.3389/fphar.2023.1150243)
Supplement: Supplementary file 1 [file DataSheet1.docx]

Supplementary Material

Table S1. Parameter values used to perform clinical trial simulations of mono-exponential increase of time-to-positivity over time to visualize the key features of each standardized pharmacometric model-based early bactericidal activity analysis approach step

| Parameter | | Description | Units | Value |
| --- | --- | --- | --- | --- |
| *BASE_TTP_* | *Cavities ≥ 4 cm* | Baseline TTP before the treatment start for different cavity extents | hours | 108^a^ |
|  | *Cavities < 4 cm* |  | hours | 131^a^ |
|  | *No cavities* |  | hours | 160^a^ |
| *SLOPE_A_* | | TTP mono-exponential rate representing the change in TTP over time for Arm A | hours/day | 0.0174^a^ |
| *SLOPE_B_* | | TTP mono-exponential rate representing the change in TTP over time for Arm B | hours/day | 0.0627^b^ |
| $\boldsymbol{SLOPE-AUC}$ | | Exponential relationship between meropenem exposure (AUC_0-inf_) and TTP slope | % change per 1 h·mg/L | 0.43^a^ |
| $\boldsymbol{IIV}_{\boldsymbol{base}}$ | | Inter-individual variability in baseline TTP | % | 6^a^ |
| $\boldsymbol{IIV}_{\boldsymbol{slope}}$ | | Inter-individual variability in TTP slope for both Arm A and Arm B | % | 22^a^ |
| $\boldsymbol{RUV}_{\boldsymbol{common}}$ | | Common residual unexplained variability for both TTP replicates | % | 13^a^ |
| $\boldsymbol{RUV}_{\boldsymbol{replicate}}$ | | Replicate-specific residual unexplained variability | % | 4^a^ |

Arm A resembling meropenem in 2 g meropenem thrice daily with 500 mg amoxicillin and 125 mg clavulanate thrice daily on days 1-14 (De Jager et al., 2022)

Arm B resembling meropenem in 6 g meropenem once daily with 2 × 1000 mg amoxicillin and 62.5 mg clavulanate plus 400 mg bedaquiline once daily on days 1-14 (unpublished data, ClinicalTrials.gov Identifier: NCT04629378)

TTP: time-to-positivity

IIV: inter-individual variability (expressed as a coefficient of variation [CV])

RUV: residual unexplained variability (expressed as coefficient of variation [CV])

^a^parameter estimate from De Jager et al. (De Jager et al., 2022)

^b^parameter estimate from unpublished data (ClinicalTrials.gov Identifier: NCT04629378)

Table S2. Parameter values of a pharmacometric early bactericidal activity model used to simulate mono-exponential increase in time-to-positivity over time to perform power calculations for different sample sizes

| Parameter | | Description | Units | Value |
| --- | --- | --- | --- | --- |
| *BASE_TTP_* | *Cavities ≥ 4 cm* | Baseline TTP before the treatment start for different cavity extents | hours | 108^a^ |
|  | *Cavities < 4 cm* |  | hours | 131^a^ |
|  | *No cavities* |  | hours | 160^a^ |
| *SLOPE_A_* | | TTP mono-exponential rate representing the change in TTP over time for Arm A | hours/day | 0.0174^a^ |
| *SLOPE_B_* | | TTP mono-exponential rate representing the change in TTP over time for Arm B | hours/day | 0.0627^b^ |
| $\boldsymbol{IIV}_{\boldsymbol{base}}$ | | Inter-individual variability in baseline TTP | % | 6^a^ |
| $\boldsymbol{IIV}_{\boldsymbol{slope,}\boldsymbol{A}}$ | | Inter-individual variability in TTP slope for Arm A | % | 104^a^ |
| $\boldsymbol{IIV}_{\boldsymbol{slope,}\boldsymbol{B}}$ | | Inter-individual variability in TTP slope for Arm B | % | 22^b^ |
| $\boldsymbol{RUV}_{\boldsymbol{common}}$ | | Common residual unexplained variability for both TTP replicates | % | 13^a^ |
| $\boldsymbol{RUV}_{\boldsymbol{replicate}}$ | | Replicate-specific residual unexplained variability | % | 4^a^ |

Arm A resembling meropenem in 2 g meropenem thrice daily with 500 mg amoxicillin and 125 mg clavulanate thrice daily on days 1-14 (De Jager et al., 2022)

Arm B resembling meropenem in 6 g meropenem once daily with 2 × 1000 mg amoxicillin and 62.5 mg clavulanate plus 400 mg bedaquiline once daily on days 1-14 (unpublished data, ClinicalTrials.gov Identifier: NCT04629378)

TTP: time-to-positivity

IIV: inter-individual variability (expressed as a coefficient of variation [CV])

RUV: residual unexplained variability (expressed as coefficient of variation [CV])

^a^parameter estimate from De Jager et al. (De Jager et al., 2022)

^b^parameter estimate from unpublished data (ClinicalTrials.gov Identifier: NCT04629378)

Table S3. Scenarios used to investigate the sample size needed to detect early bactericidal activity (EBA) using time-to-positivity (TTP) and a pharmacometric model-based analysis for different time-to-positivity slope (SLOPE_TTP_) values and low and high inter-individual variability (IIV) in EBA

| TTP-EBA model | TTP-EBA_0-14_  [SLOPE_TTP_] | IIV_SLOPE_ | Scenarios |
| --- | --- | --- | --- |
| BASE_TTP_  Cavities ≥ 4 cm = 108 hours^a^  Cavities < 4 cm = 131 hours^a^  No cavities = 160 hours^a^  IIV_base_ = 6%^a^  RUV_common_ = 13%^a^  RUV_replicate_ = 4%^a^ | 152 hours  [0.0628 hours/day]^b^ | 22%^b^ | TTP-EBA_0-14_ 152 hours, IIV_SLOPE_ 22% |
|  |  | 104%^a^ | TTP-EBA_0-14_ 152 hours, IIV_SLOPE_ 104% |
|  | 30 hours  [0.0174 hours/day]^a^ | 22%^b^ | TTP-EBA_0-14_ 30 hours, IIV_SLOPE_ 22% |
|  |  | 104%^a^ | TTP-EBA_0-14_ 30 hours, IIV_SLOPE_ 104% |
|  | 25 hours  [0.0148 hours/day] | 22%^b^ | TTP-EBA_0-14_ 25 hours, IIV_SLOPE_ 22% |
|  |  | 104%^a^ | TTP-EBA_0-14_ 25 hours, IIV_SLOPE_ 104% |
|  | 20 hours  [0.0122] | 22%^b^ | TTP-EBA_0-14_ 20 hours, IIV_SLOPE_ 22% |
|  |  | 104%^a^ | TTP-EBA_0-14_ 20 hours, IIV_SLOPE_ 104% |
|  | 16 hours  [0.0098 hours/day] | 22%^b^ | TTP-EBA_0-14_ 16 hours, IIV_SLOPE_ 22% |
|  |  | 104%^a^ | TTP-EBA_0-14_ 16 hours, IIV_SLOPE_ 104% |
|  | 11 hours  [0.0069 hours/day] | 22%^b^ | TTP-EBA_0-14_ 11 hours, IIV_SLOPE_ 22% |
|  |  | 104%^a^ | TTP-EBA_0-14_ 11 hours, IIV_SLOPE_ 104% |
|  | 7 hours  [0.0044 hours/day] | 22%^b^ | TTP-EBA_0-14_ 7 hours, IIV_SLOPE_ 22% |
|  |  | 104%^a^ | TTP-EBA_0-14_ 7 hours, IIV_SLOPE_ 104% |
|  | 3 hours  [0.0017 hours/day] | 22%^b^ | TTP-EBA_0-14_ 3 hours, IIV_SLOPE_ 22% |
|  |  | 104%^a^ | TTP-EBA_0-14_ 3 hours, IIV_SLOPE_ 104% |

Mono-exponential function with proportional RUV was used to simulate TTP over time. BASE_TTP_, IIV_base_, RUV_common_ and RUV_replicate_ were the same in all scenarios.

EBA was expressed as early bactericidal activity based on difference in time-to-positivity between 0 and 14 days (TTP-EBA_0-14_)

IIV: inter-individual variability expressed as a coefficient of variation (CV)

RUV: residual unexplained variability, a proportional error model was used with both common error (RUV_common_) and replicate-specific (RUV_replicate_) error terms for each replicate

h: hours

^a^parameter estimate from De Jager et al. (De Jager et al., 2022)

^b^parameter estimate from unpublished data (ClinicalTrials.gov Identifier: NCT04629378)

Table S4. Scenarios used to investigate the sample size needed to detect a difference in early bactericidal activity (EBA) between treatment arms using time-to-positivity (TTP) and a model-based analysis for different time-to-positivity slope (SLOPE_TTP_) values and low and high inter-individual variability (IIV) in EBA

| TTP-EBA_0-14_  [SLOPE_TTP_] | IIV_SLOPE_ | Effect difference^*^ | Scenarios |
| --- | --- | --- | --- |
| *TTP-EBA_0-14_ of 152 hours and IIV in TTP slope of 22% CV* | | | |
| 152 hours  [0.0628 hours/day]^a^ | 22%^a^ | 25% | TTP-EBA_0-14_ 152 hours, IIV_SLOPE_ 22%, effect difference 25% |
| 152 hours  [0.0628 hours/day]^a^ | 22%^a^ | 50% | TTP-EBA_0-14_ 152 hours, IIV_SLOPE_ 22%, effect difference 50% |
| 152 hours  [0.0628 hours/day]^a^ | 22%^a^ | 75% | TTP-EBA_0-14_ 152 hours, IIV_SLOPE_ 22%, effect difference 75% |
| 152 hours  [0.0628 hours/day]^a^ | 22%^a^ | 100% | TTP-EBA_0-14_ 152 hours, IIV_SLOPE_ 22%, effect difference 100% |
| 152 hours  [0.0628 hours/day]^a^ | 22%^a^ | 125% | TTP-EBA_0-14_ 152 hours, IIV_SLOPE_ 22%, effect difference 125% |
| 152 hours  [0.0628 hours/day]^a^ | 22%^a^ | 150% | TTP-EBA_0-14_ 152 hours, IIV_SLOPE_ 22%, effect difference 150% |
| 152 hours  [0.0628 hours/day]^a^ | 22%^a^ | 175% | TTP-EBA_0-14_ 152 hours, IIV_SLOPE_ 22%, effect difference 175% |
| 152 hours  [0.0628 hours/day]^a^ | 22%^a^ | 200% | TTP-EBA_0-14_ 152 hours, IIV_SLOPE_ 22%, effect difference 200% |

| TTP-EBA_0-14_ *of 152 hours and IIV in TTP slope of 104% CV* | | | |
| --- | --- | --- | --- |
| 152 hours  [0.0628 hours/day]^a^ | 104%^b^ | 25% | TTP-EBA_0-14_ 152 hours, IIV_SLOPE_ 104%, effect difference 25% |
| 152 hours  [0.0628 hours/day]^a^ | 104%^b^ | 50% | TTP-EBA_0-14_ 152 hours, IIV_SLOPE_ 104%, effect difference 50% |
| 152 hours  [0.0628 hours/day]^a^ | 104%^b^ | 75% | TTP-EBA_0-14_ 152 hours, IIV_SLOPE_ 104%, effect difference 75% |
| 152 hours  [0.0628 hours/day]^a^ | 104%^b^ | 100% | TTP-EBA_0-14_ 152 hours, IIV_SLOPE_ 104%, effect difference 100% |
| 152 hours  [0.0628 hours/day]^a^ | 104%^b^ | 125% | TTP-EBA_0-14_ 152 hours, IIV_SLOPE_ 104%, effect difference 125% |
| 152 hours  [0.0628 hours/day]^a^ | 104%^b^ | 150% | TTP-EBA_0-14_ 152 hours IIV_SLOPE_ 104%, effect difference 150% |
| 152 hours  [0.0628 hours/day]^a^ | 104%^b^ | 175% | TTP-EBA_0-14_ 152 hours, IIV_SLOPE_ 104%, effect difference 175% |
| 152 hours  [0.0628 hours/day]^a^ | 104%^b^ | 200% | TTP-EBA_0-14_ 152 hours, IIV_SLOPE_ 104%, effect difference 200% |

| TTP-EBA_0-14_ *of 30 hours and IIV in TTP slope of 22% CV* | | | |
| --- | --- | --- | --- |
| 30 hours  [0.0174 hours/day]^b^ | 22%^a^ | 25% | TTP-EBA_0-14_ 30 hours, IIV_SLOPE_ 22%, effect difference 25% |
| 30 hours  [0.0174 hours/day]^b^ | 22%^a^ | 50% | TTP-EBA_0-14_ 30 hours, IIV_SLOPE_ 22%, effect difference 50% |
| 30 hours  [0.0174 hours/day]^b^ | 22%^a^ | 75% | TTP-EBA_0-14_ 30 hours, IIV_SLOPE_ 22%, effect difference 75% |
| 30 hours  [0.0174 hours/day]^b^ | 22%^a^ | 100% | TTP-EBA_0-14_ 30 hours IIV_SLOPE_ 22%, effect difference 100% |
| 30 hours  [0.0174 hours/day]^b^ | 22%^a^ | 125% | TTP-EBA_0-14_ 30 hours, IIV_SLOPE_ 22%, effect difference 125% |
| 30 hours  [0.0174 hours/day]^b^ | 22%^a^ | 150% | TTP-EBA_0-14_ 30 hours, IIV_SLOPE_ 22%, effect difference 150% |
| 30 hours  [0.0174 hours/day]^b^ | 22%^a^ | 175% | TTP-EBA_0-14_ 30 hours, IIV_SLOPE_ 22%, effect difference 175% |
| 30 hours  [0.0174 hours/day]^b^ | 22%^a^ | 200% | TTP-EBA_0-14_ 30 hours, IIV_SLOPE_ 22%, effect difference 200% |

| TTP-EBA_0-14_ *of 30 hours and IIV TTP slope 104% CV* | | | |
| --- | --- | --- | --- |
| 30 hours  [0.0174 hours/day]^b^ | 104%^b^ | 25% | TTP-EBA_0-14_ 30 hours, IIV_SLOPE_ 104%, effect difference 25% |
| 30 hours  [0.0174 hours/day]^b^ | 104%^b^ | 50% | TTP-EBA_0-14_ 30 hours, IIV_SLOPE_ 104%, effect difference 50% |
| 30 hours  [0.0174 hours/day]^b^ | 104%^b^ | 75% | TTP-EBA_0-14_ 30 hours, IIV_SLOPE_ 104%, effect difference 75% |
| 30 hours  [0.0174 hours/day]^b^ | 104%^b^ | 100% | TTP-EBA_0-14_ 30 hours, IIV_SLOPE_ 104%, effect difference 100% |
| 30 hours  [0.0174 hours/day]^b^ | 104%^b^ | 125% | TTP-EBA_0-14_ 30 hours, IIV_SLOPE_ 104%, effect difference 125% |
| 30 hours  [0.0174 hours/day]^b^ | 104%^b^ | 150% | TTP-EBA_0-14_ 30 hours, IIV_SLOPE_ 104%, effect difference 150% |
| 30 hours  [0.0174 hours/day]^b^ | 104%^b^ | 175% | TTP-EBA_0-14_ 30 hours, IIV_SLOPE_ 104%, effect difference 175% |
| 30 hours  [0.0174 hours/day]^b^ | 104%^b^ | 200% | TTP-EBA_0-14_ 30 hours, IIV_SLOPE_ 104%, effect difference 200% |

^*^Effect difference was expressed as the percentage higher TTP slope between the two treatment arms

Mono-exponential function with proportional RUV was used to simulate TTP over time. BASE_TTP_, IIV_base_, RUV_common_ and RUV_replicate_ were the same in all scenarios.

EBA was expressed as early bactericidal activity based on difference in time-to-positivity between 0 and 14 days (TTP-EBA_0-14_)

IIV: inter-individual variability expressed as a coefficient of variation (CV)

RUV: residual unexplained variability, a proportional error model was used with both common error (RUV_common_ ) and replicate-specific error (RUV_replicate_) terms for each replicate

h: hours

^a^parameter estimate from unpublished data (ClinicalTrials.gov Identifier: NCT04629378)

^b^parameter estimate from De Jager et al. (De Jager et al., 2022)

Table S5. Scenarios used to investigate the sample size needed to detect a difference in early bactericidal activity (EBA) between two treatment groups using time-to-positivity (TTP) and a model-based analysis with different inter-individual variability (IIV) in TTP slope

| TTP-EBA_0-14_ [SLOPE_TTP_] | IIV_SLOPE_ | Effect difference^*^ | Scenarios |
| --- | --- | --- | --- |
| 152 hours  [0.0628 hours/day]^a^ | 10% | 50% | TTP-EBA_0-14_ 152 hours, IIV_SLOPE_ 10%, effect difference 50% |
| 152 hours  [0.0628 hours/day]^a^ | 22%^a^ | 50% | TTP-EBA_0-14_ 152 hours, IIV_SLOPE_ 22%, effect difference 50% |
| 152 hours  [0.0628 hours/day]^a^ | 40% | 50% | TTP-EBA_0-14_ 152 hours, IIV_SLOPE_ 40%, effect difference 50% |
| 152 hours  [0.0628 hours/day]^a^ | 60% | 50% | TTP-EBA_0-14_ 152 hours, IIV_SLOPE_ 60%, effect difference 50% |
| 152 hours  [0.0628 hours/day]^a^ | 80% | 50% | TTP-EBA_0-14_ 152 hours, IIV_SLOPE_ 80%, effect difference 50% |
| 152 hours  [0.0628 hours/day]^a^ | 104%^b^ | 50% | TTP-EBA_0-14_ 152 hours, IIV_SLOPE_ 104%, effect difference 50% |
| 30 hours  [0.0174 hours/day]^b^ | 10% | 50% | TTP-EBA_0-14_ 30 hours, IIV_SLOPE_ 10%, effect difference 50% |
| 30 hours  [0.0174 hours/day]^b^ | 22%^a^ | 50% | TTP-EBA_0-14_ 30 hours, IIV_SLOPE_ 22%, effect difference 50% |
| 30 hours  [0.0174 hours/day]^b^ | 40% | 50% | TTP-EBA_0-14_ 30 hours, IIV_SLOPE_ 40%, effect difference 50% |
| 30 hours  [0.0174 hours/day]^b^ | 60% | 50% | TTP-EBA_0-14_ 30 hours, IIV_SLOPE_ 60%, effect difference 50% |
| 30 hours  [0.0174 hours/day]^b^ | 80% | 50% | TTP-EBA_0-14_ 30 hours, IIV_SLOPE_ 80%, effect difference 50% |
| 30 hours  [0.0174 hours/day]^b^ | 104%^b^ | 50% | TTP-EBA_0-14_ 30 hours, IIV_SLOPE_ 104%, effect difference 50% |

^*^Effect difference was expressed as the percentage higher TTP slope between two treatment arms

Mono-exponential function with proportional RUV was used to simulate TTP over time. BASE_TTP_, IIV_base_, RUV_common_ and RUV_replicate_ were the same in all scenarios.

EBA was expressed as early bactericidal activity based on difference in time-to-positivity between 0 and 14 days (TTP-EBA_0-14_)

IIV: inter-individual variability expressed as a coefficient of variation (CV)

RUV: residual unexplained variability, a proportional error model was used with both common and replicate-specific error terms for each replicate

h: hours

^a^parameter estimate from unpublished data (ClinicalTrials.gov Identifier: NCT04629378)

^b^parameter estimate from De Jager et al. (De Jager et al., 2022)

Table S6. Summary statistics of covariate distributions for treatment arms A and B as well as for all patients, based on the simulated data to visualize the standardized pharmacometric model-based early bactericidal activity (EBA) analysis approach

|  |  | Arm A | Arm B | All |
| --- | --- | --- | --- | --- |
| Participants | *Number* | 15 | 15 | 30 |
| Cavitary disease | *No* | 5 (33.33%) | 5 (33.33%) | 15 (33.33%) |
|  | *Yes (< 4 cm)* | 5 (33.33%) | 5 (33.33%) | 15 (33.33%) |
|  | *male* | 5 (33.33%) | 5 (33.33%) | 15 (33.33%) |
| Sex | *Male* | 8 (53.33%) | 7 (46.67%) | 15 (50%) |
|  | *Female* | 7 (46.67%) | 8 (53.33%) | 15 (50%) |
| Meropenem AUC (h∙mg/L) | *Median* | 666.34 | 649.39 | 653.85 |
|  | *IQR* | 584.35-694.51 | 610.81-710.66 | 589.49-700.85 |
|  | *Min-max* | 447.54-775.19 | 540.79-808.66 | 447.54-808.66 |
| Age (years) | *Median* | 38 | 39 | 39 |
|  | *IQR* | 32-43.5 | 35-45 | 33-44 |
|  | *Min-max* | 27-51 | 25-58 | 25-58 |

Arm A resembling 2 g meropenem thrice daily with 500 mg amoxicillin and 125 mg clavulanate thrice daily on days 1-14 (De Jager et al., 2022)

Arm B resembling 6 g meropenem once daily with 2 × 1000 mg amoxicillin and 62.5 mg clavulanate plus 400 mg bedaquiline once daily on days 1-14 (unpublished data, ClinicalTrials.gov Identifier: NCT04629378)

AUC: area under the concentration versus time curve

IQR: interquartile range

Max: maximum

Min: minimum

Table S7. Non-positive and censored (negative at day 42) data in the simulated dataset for time-to-positivity (TTP) at the different time points used to visualize the standardized pharmacometric model-based early bactericidal activity (EBA) analysis approach

| Sample status | Day 0 | Day 1 | Day 2 | Day 3 | Day 4 | Day 6 | Day 8 | Day 10 | Day 12 | Day 14 |
| --- | --- | --- | --- | --- | --- | --- | --- | --- | --- | --- |
| Contaminated | 0 | 1 | 3 | 0 | 1 | 1 | 3 | 2 | 1 | 0 |
| Negative at day 42 | 1 | 0 | 3 | 1 | 0 | 2 | 0 | 2 | 1 | 2 |
| No result | 1 | 0 | 1 | 2 | 0 | 2 | 3 | 0 | 1 | 2 |
| Not done | 0 | 1 | 2 | 0 | 1 | 2 | 2 | 2 | 0 | 2 |

Table S8. Parameter estimates of the final pharmacometric early bacterial activity time-to-positivity (EBA-TTP) model based on simulated TTP data to visualize the standardized pharmacometric model-based EBA analysis approach

| Parameter | | Description | Units | Parameter value | RSE (%) | Shrinkage (%) |
| --- | --- | --- | --- | --- | --- | --- |
| *BASE_TTP_* | ***Cavities ≥ 4 cm*** | Baseline TTP for different cavitary disease states | hours | 98 | 19 | - |
|  | ***Cavities < 4 cm*** |  | hours | 122 | 43 | - |
|  | ***No cavities*** |  | hours | 153 | 1 | - |
| *SLOPE_A_* | | TTP mono-exponential rate representing change in TTP over time for Arm A | hours/day | 0.0187 | 12 | - |
| *SLOPE_B_* | | TTP mono-exponential rate representing change in TTP over time for Arm B | hours/day | 0.0696 | 5 | - |
| *COV_SLOPE-AUC_* | | Drug exposure covariate on TTP slope expressed as a percentage change in slope per 1 h∙mg/L | % | 0.44 | 5 | - |
| $\boldsymbol{IIV}_{\boldsymbol{baseline}}$ | | Inter-individual variability in TTP baseline | % | 4.3 | 9 | 1 |
| $\boldsymbol{IIV}_{\boldsymbol{slope}}$ | | Inter-individual variability in TTP slope | % | 20.7 | 27 | 32 |
| $\boldsymbol{RUV}_{\boldsymbol{common}}$ | | Common residual unexplained variability for both TTP replicates | % | 11.7 | 4 | 4 |
| $\boldsymbol{RUV}_{\boldsymbol{replicate}}$ | | Replicate-specific residual unexplained variability | % | 4 | 4 | 3 |

Arm A resembling 2 g meropenem thrice daily with 500 mg amoxicillin and 125 mg clavulanate thrice daily on days 1-14 (De Jager et al., 2022)

Arm B resembling 6 g meropenem once daily with 2 × 1000 mg amoxicillin and 62.5 mg clavulanate plus 400 mg bedaquiline once daily on days 1-14 (unpublished data, ClinicalTrials.gov Identifier: NCT04629378)

RSE: relative standard error

TTP: time-to-positivity

IIV: inter-individual variability (expressed as a coefficient of variation [CV])

RUV: residual unexplained variability (expressed as coefficient of variation [CV])

Table S9. Model-based typical predictions of the early bactericidal activity (EBA) for 0-2, 0-7, and 0-14 days in time-to-positivity (TTP), based on the simulated data to visualize the standardized pharmacometric model-based EBA analysis approach.

|  | TTP-EBA_0-2_ | TTP-EBA_0-7_ | TTP-EBA_0-14_ |
| --- | --- | --- | --- |
| Arm A | 4 hours | 14 hours | 29 hours |
| Arm B | 15 hours | 61 hours | 161 hours |

Typical individual with cavities ≥ 4 cm

TTP-EBA_0-XX_: difference in TTP between day 0 and day XX (where XX is 2, 7 or 14)

Arm A resembling 2 g meropenem thrice daily with 500 mg amoxicillin and 125 mg clavulanate thrice daily on days 1-14 (De Jager et al., 2022)

Arm B resembling 6 g meropenem once daily with 2 × 1000 mg amoxicillin and 62.5 mg clavulanate plus 400 mg bedaquiline once daily on days 1-14 (unpublished data, ClinicalTrials.gov Identifier: NCT04629378)

Table S10. Model-based predicted median and 2.5-97.5^th^ percentiles of individual early bactericidal activity (EBA) for 0-2, 0-7, and 0-14 days in TTP, based on Bayes estimates of the final time-to-positivity (TTP)-EBA model, based on the simulated data to visualize the standardized pharmacometric model-based EBA analysis approach

|  | TTP-EBA_0-2_ | TTP-EBA_0-7_ | TTP-EBA_0-14_ |
| --- | --- | --- | --- |
| Arm A | 5 (2 – 7) hours | 20 (8 – 26) hours | 42 (18 – 59) hours |
| Arm B | 18 (3 – 39) hours | 79 (12 – 187) hours | 223 (28 – 612) hours |

TTP-EBA_0-XX_: difference in TTP between day 0 and day XX (where XX is 2, 7 or 14)

Arm A resembling 2 g meropenem thrice daily with 500 mg amoxicillin and 125 mg clavulanate thrice daily on days 1-14 (De Jager et al., 2022)

Arm B resembling 6 g meropenem once daily with 2 × 1000 mg amoxicillin and 62.5 mg clavulanate plus 400 mg bedaquiline once daily on days 1-14 (unpublished data, ClinicalTrials.gov Identifier: NCT04629378)


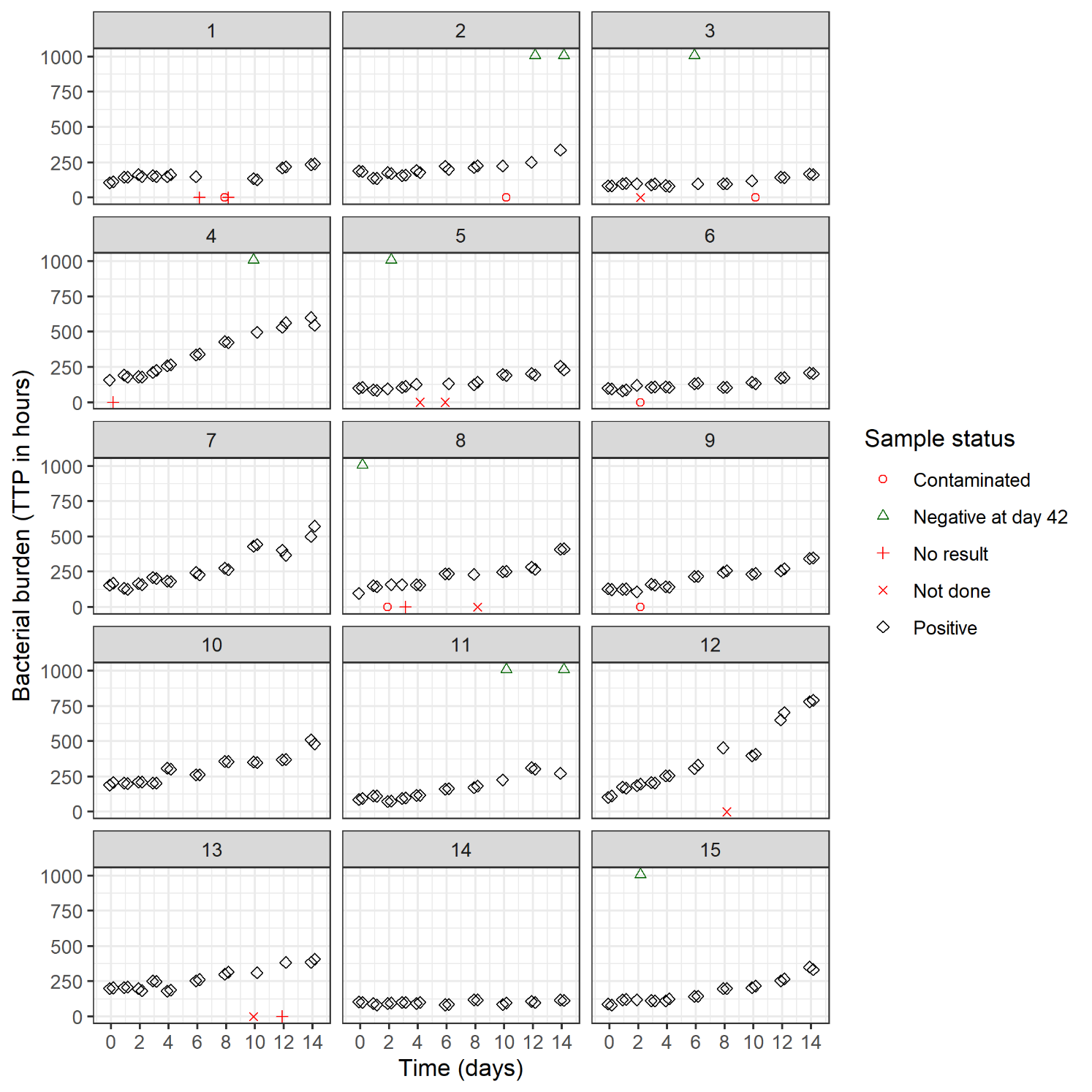


Figure S1. Illustration of individual time-to-positivity (TTP) biomarker versus time. Both replicates 1 and 2 for each timepoint are included. The graphical analysis aims to identify observations where the two replicates do not overlap which would indicate a reason to omit observation. A non-positive replicate is plotted in red (missing) or green (negative) on the x-axis with the shape giving the reason for its non-positive sample status. These symbols are plotted next to each other to facilitate interpretation but belong to the same timepoint. Plots are based on the simulated data to visualize the standardized pharmacometric model-based early bactericidal activity (EBA) analysis approach.


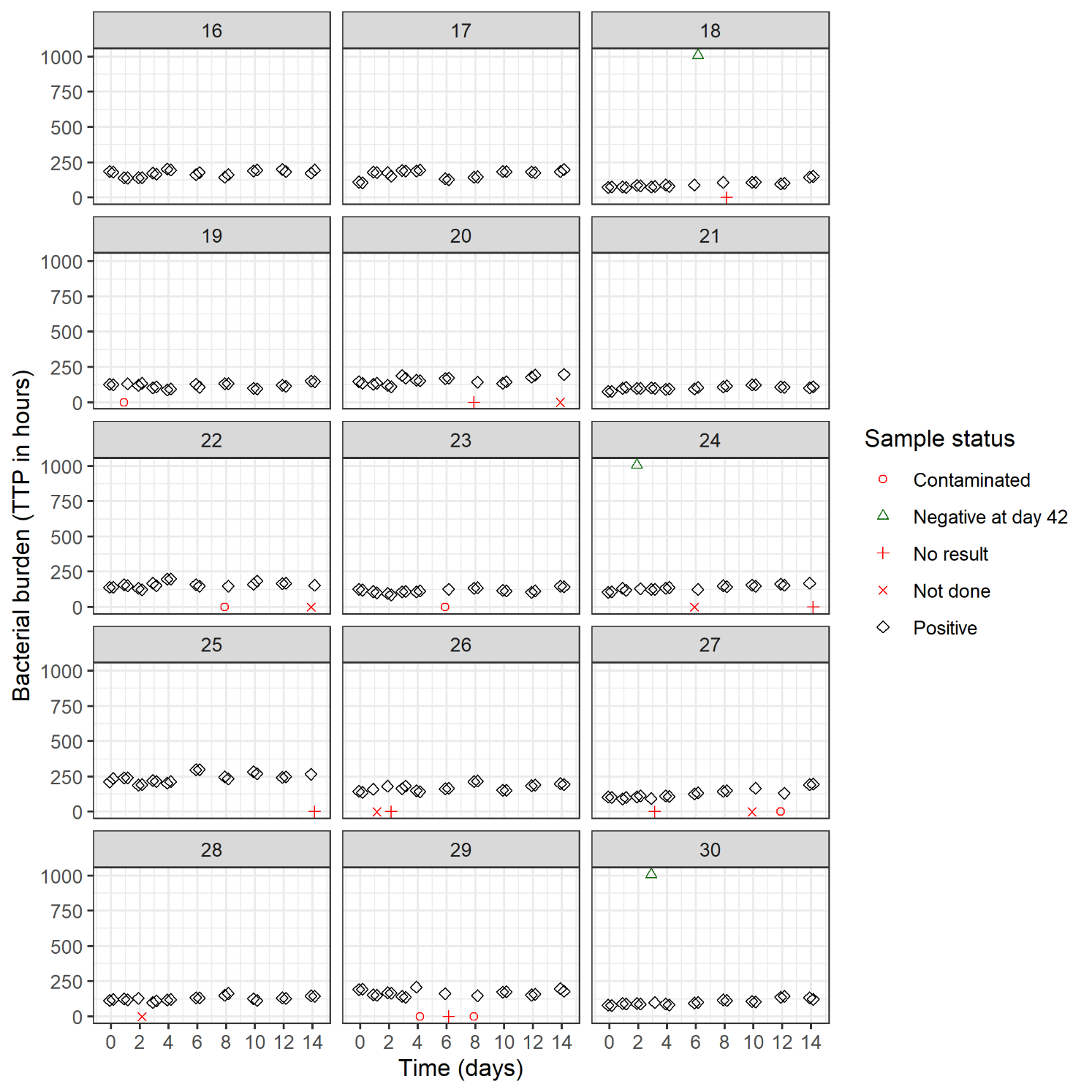


Figure S1. Illustration of individual time-to-positivity (TTP) biomarker versus time. Both replicates 1 and 2 for each timepoint are included. The graphical analysis aims to identify observations where the two replicates do not overlap which would indicate a reason to omit observation. A non-positive replicate is plotted in red (missing) or green (negative) on the x-axis with the shape giving the reason for its non-positive sample status. These symbols are plotted next to each other to facilitate interpretation but belong to the same timepoint. Plots are based on the simulated data to visualize the standardized pharmacometric model-based early bactericidal activity (EBA) analysis approach.


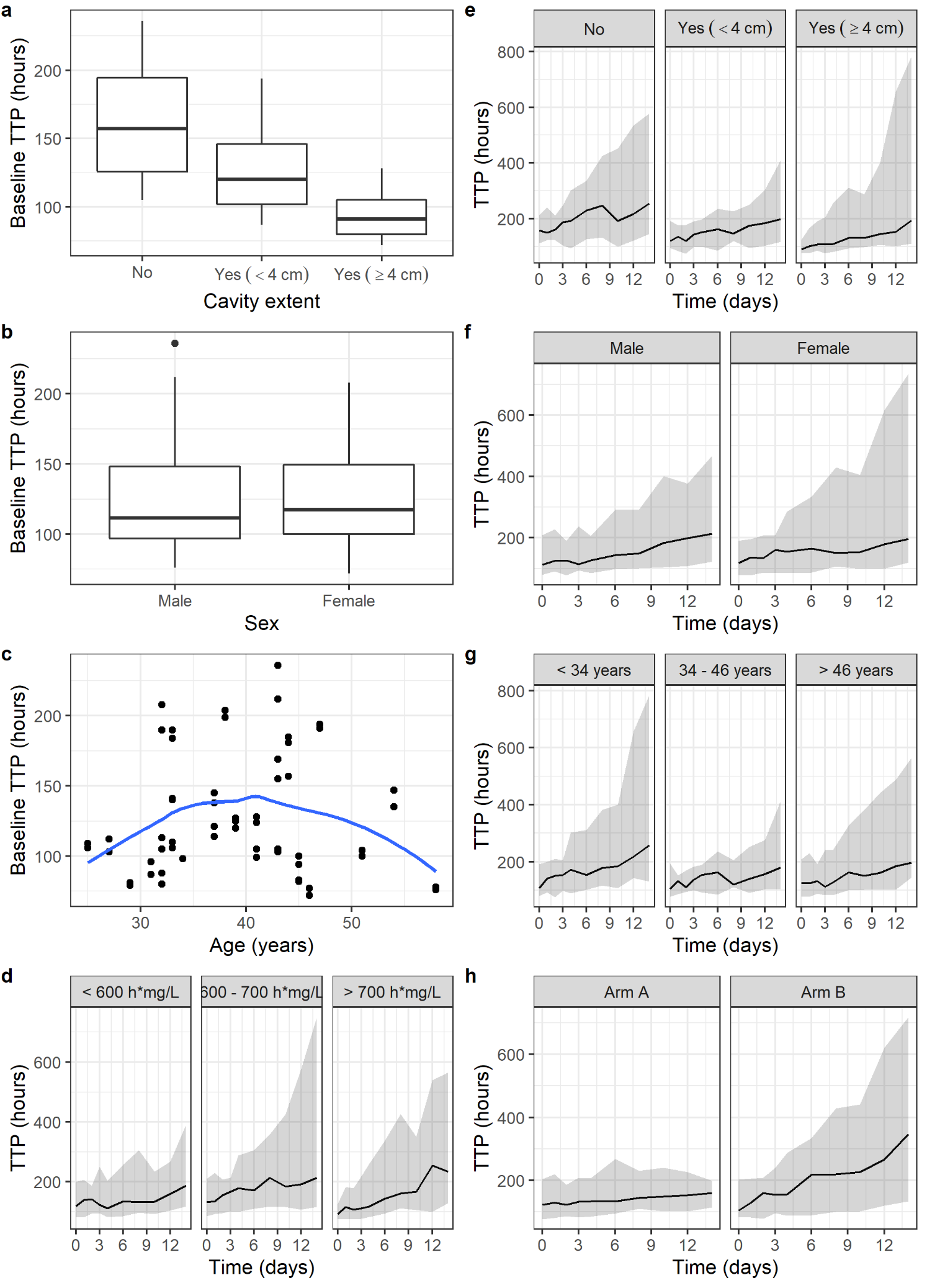


Figure S2. Illustration of graphical analysis plots with baseline time-to-positivity (TTP) versus cavity extent (a), sex (b) and age (c), and TTP over time stratified by drug exposure (d), cavity extent (e), sex (f), age (g) and treatment arm (h). Plots are based on the simulated data to visualize the standardized pharmacometric model-based early bactericidal activity (EBA) analysis approach.

Boxplot horizontal line represents the median while hinges correspond to first and third quartiles, whiskers extend to the largest or smallest value no further than 150% of the interquartile range from the hinge, and outliers beyond the whiskers are shown as symbols. Shaded areas represent 5-95^th^ quantiles of observed data with the observed median as a solid line. Observations are shown as symbols with a loess smooth shown as a blue line.

Arm A: treatment arm resembling 2 g meropenem thrice daily with 500 mg amoxicillin and 125 mg clavulanate thrice daily on days 1-14 (De Jager et al., 2022)

Arm B: treatment arm resembling 6 g meropenem once daily with 2 × 1000 mg amoxicillin and 62.5 mg clavulanate plus 400 mg bedaquiline once daily on days 1-14 (unpublished data, ClinicalTrials.gov Identifier: NCT04629378)


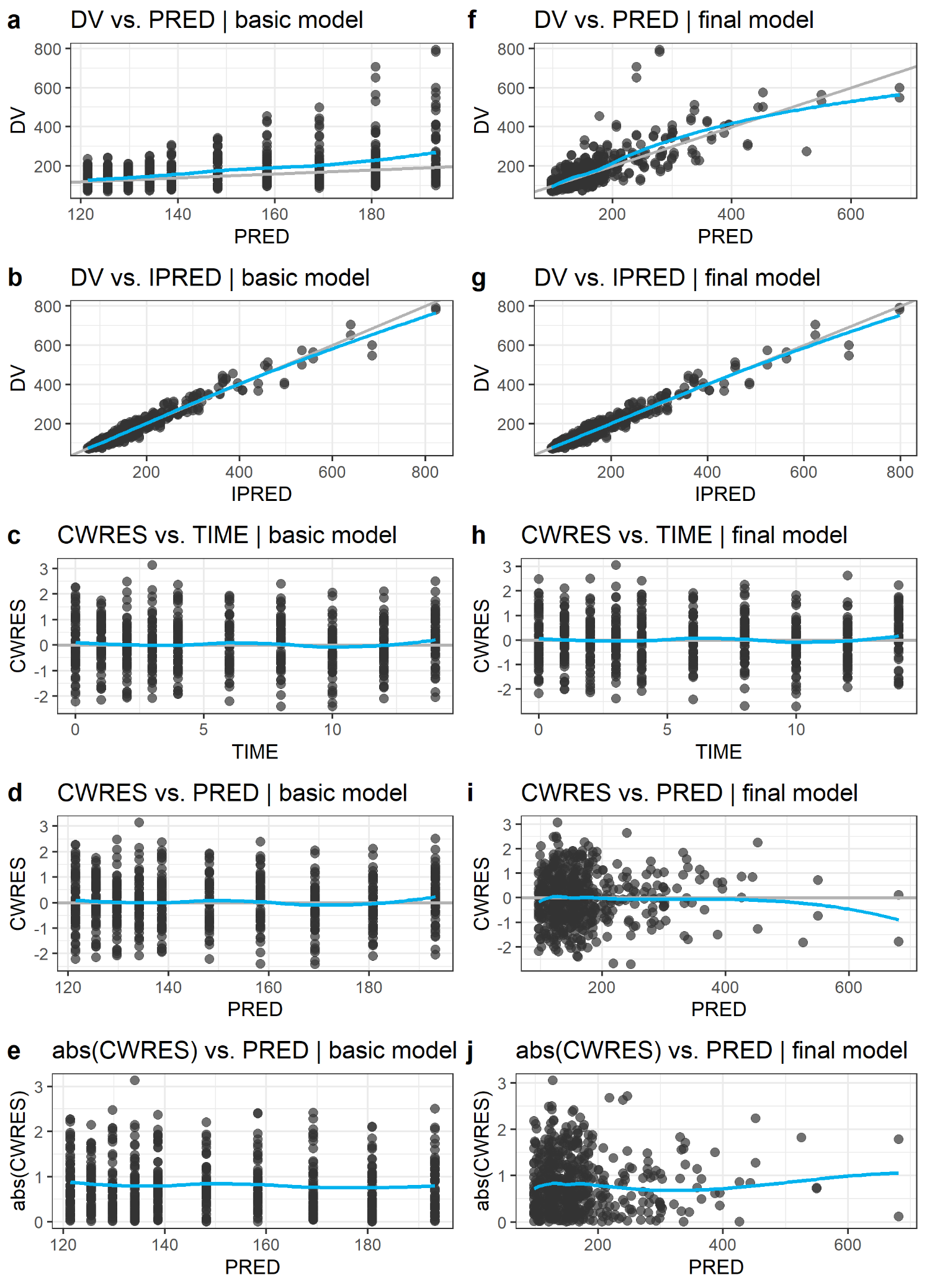


Figure S3. Illustration of goodness-of-fit plots of a base (plots a-e) and a final (f-j) early bactericidal activity time-to-positivity (EBA-TTP) model. Plots are based on the simulated data to visualize the standardized pharmacometric model-based EBA analysis approach.

Panels a and f: population predictions versus observations for a base and a final model. Grey line represents the identity line, while the blue line is smoothed trendline throughout the data. Overlap of identity and trendline indicates a good model fit.

Panels b and g: individual predictions versus observations for a base and a final model. Grey line represents the identity line, while the blue line is smoothed trendline throughout the data. Overlap of identity and trendline indicates a good model fit.

Panels c and h: conditional weighted residuals (CWRES) versus time for a base and a final model. CWRES are expected to be distributed around zero with a spread mainly between -2 and 2. A trendless blue loess smooth line indicates that the model describes data well over time.

Panels d and i: conditional weighted residuals (CWRES) versus population predictions for a base and a final model. CWRES are expected to be distributed around zero with a spread mainly between -2 and 2. A trendless blue loess smooth line indicates that no model misspecification is present.

Panels e and j: absolute conditional individual weighted residuals [abs(CWRES)] versus population predictions (PRED) for a base and a final model. A trendless blue loess smooth line indicates that an appropriate residual unexplained variability model was selected.


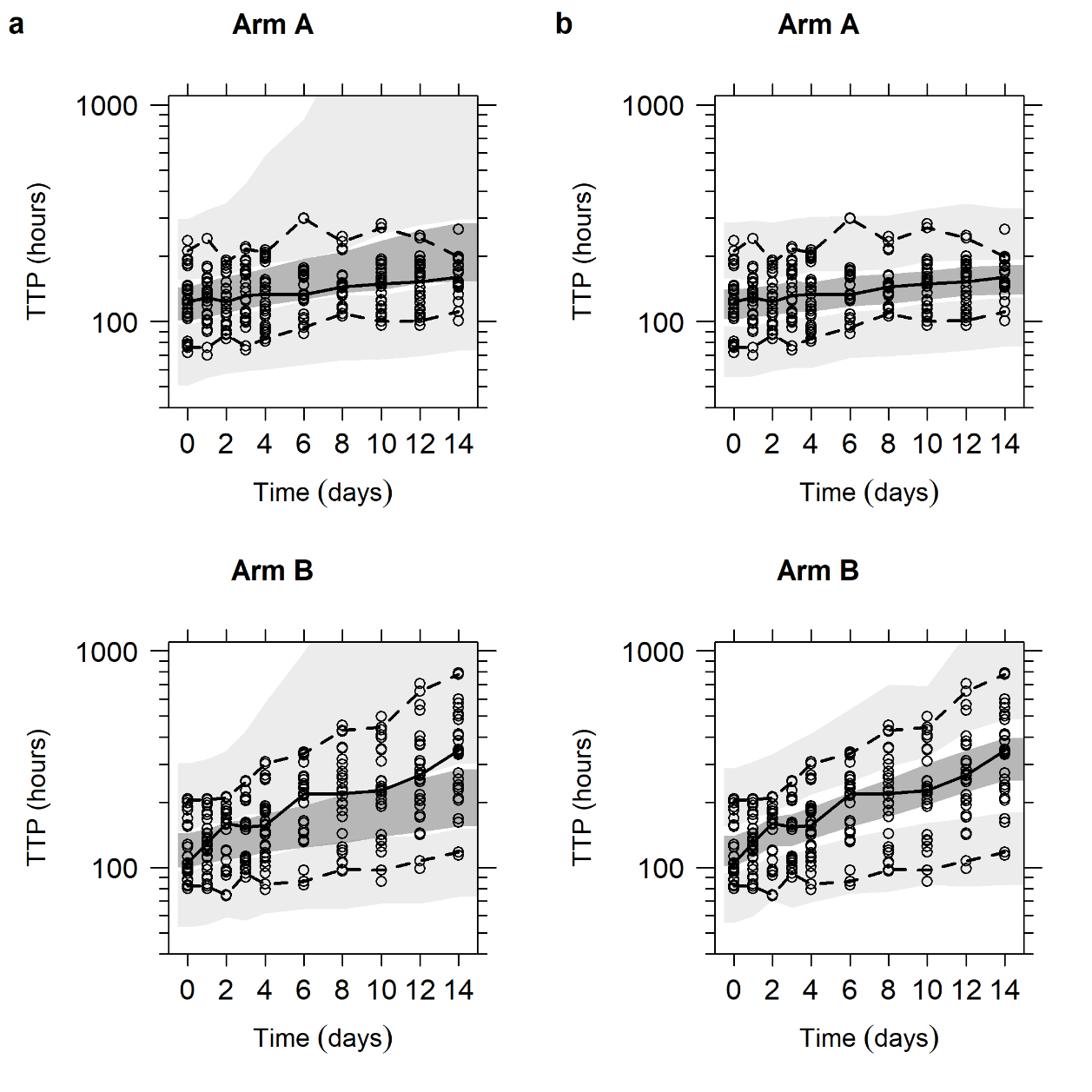


Figure S4. Illustration of visual predictive check (VPC) for an early bactericidal activity time-to-positivity (EBA-TTP) model stratified on treatment group for a) base model, b final model. Solid and dashed lines represent the median and 2.5^th^ and 97.5^th^ percentiles of the observed data, respectively. Shaded areas represent 95% confidence intervals of the 97.5^th^, median and 2.5^th^ percentiles of the simulated data from 1000 simulations. Circles represent observations. Plots are based on the simulated data to visualize the standardized pharmacometric model-based EBA analysis approach.

Arm A: treatment arm resembling 2 g meropenem thrice daily with 500 mg amoxicillin and 125 mg clavulanate thrice daily on days 1-14 (De Jager et al., 2022)

Arm B: treatment arm resembling 6 g meropenem once daily with 2 × 1000 mg amoxicillin and 62.5 mg clavulanate plus 400 mg bedaquiline once daily on days 1-14 (unpublished data, ClinicalTrials.gov Identifier: NCT04629378)
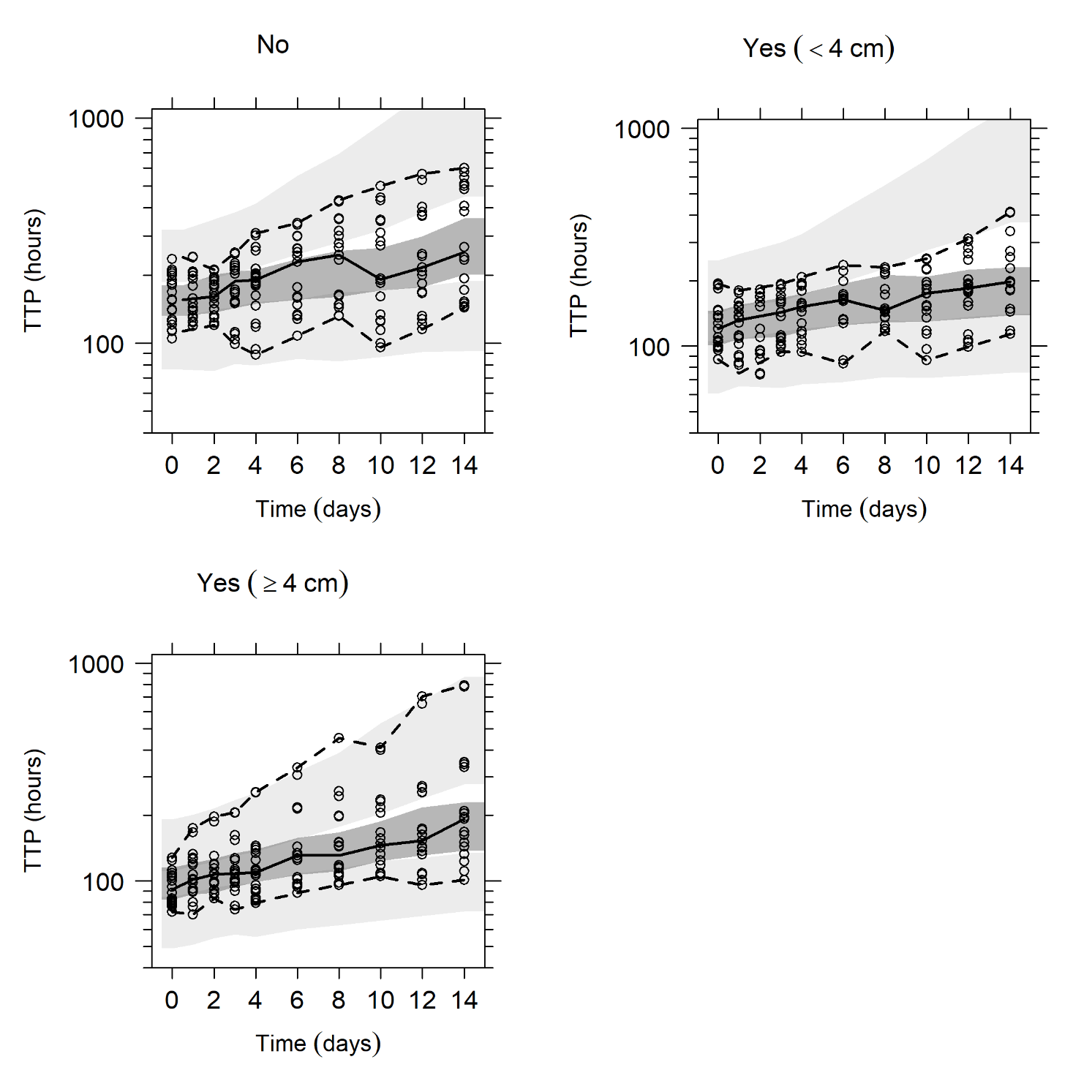
Figure S5. Illustration of a visual predictive check (VPC) stratified on the categorical covariate cavity extent based on an early bactericidal activity time-to-positivity (EBA-TTP) model. Solid and dashed lines represent the median and 2.5^th^ and 97.5^th^ percentiles of the observed data, respectively. Shaded areas represent 95% confidence intervals of the 97.5^th^, median and 2.5^th^ percentiles of the simulated data from 1000 simulations. Circles represent observations. Plots are based on the simulated data to visualize the standardized pharmacometric model-based EBA analysis approach.


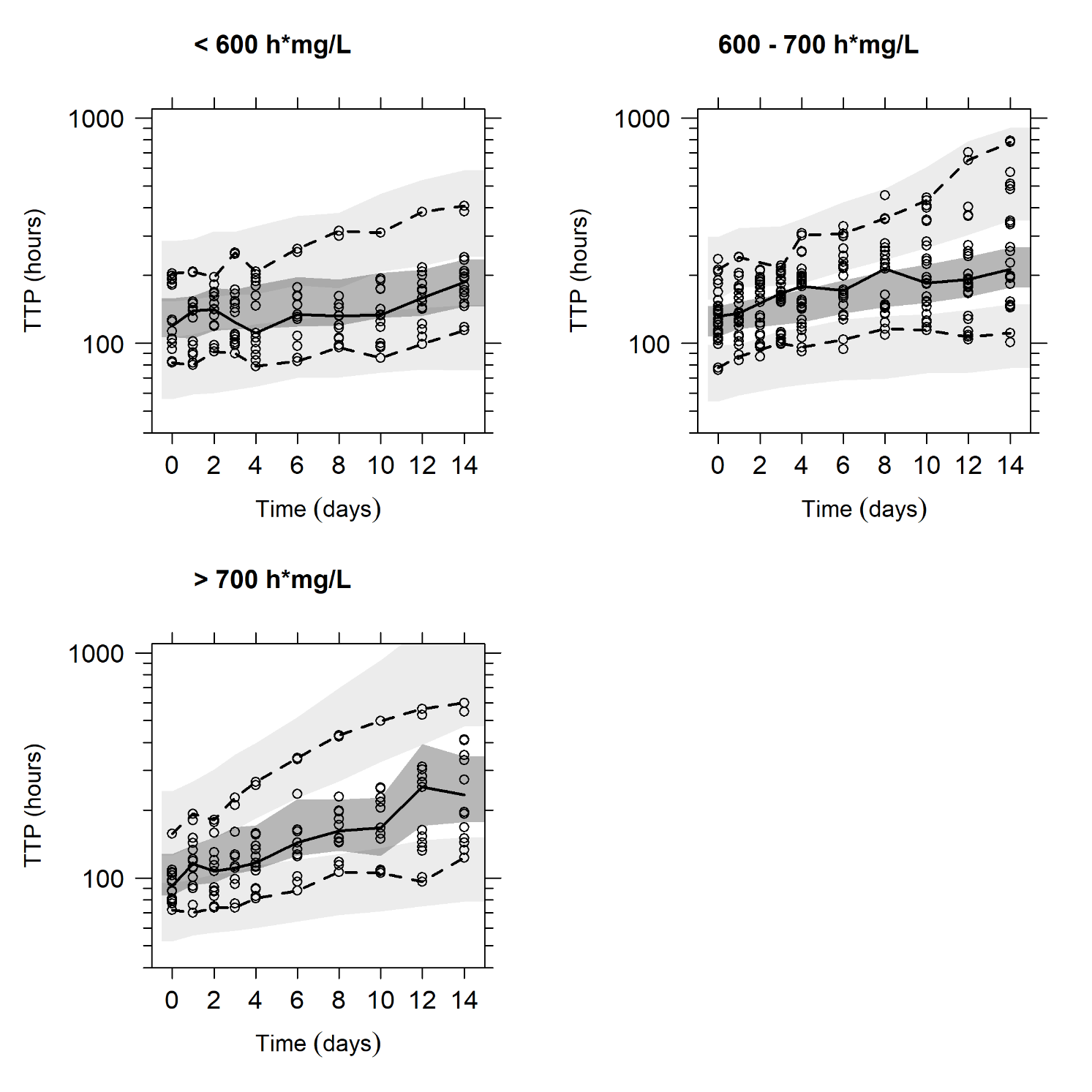
Figure S6. Illustration of a visual predictive check (VPC) stratified on the continuous covariate area under the concentration versus time curve (AUC) based on an early bactericidal activity time-to-positivity (EBA-TTP) model. Solid and dashed lines represent the median and 2.5^th^ and 97.5^th^ percentiles of the observed data, respectively. Shaded areas represent 95% confidence intervals of the 97.5^th^, median and 2.5^th^ percentiles of the simulated data from 1000 simulations. Circles represent observations. Plots are based on the simulated data to visualize the standardized pharmacometric model-based EBA analysis approach.


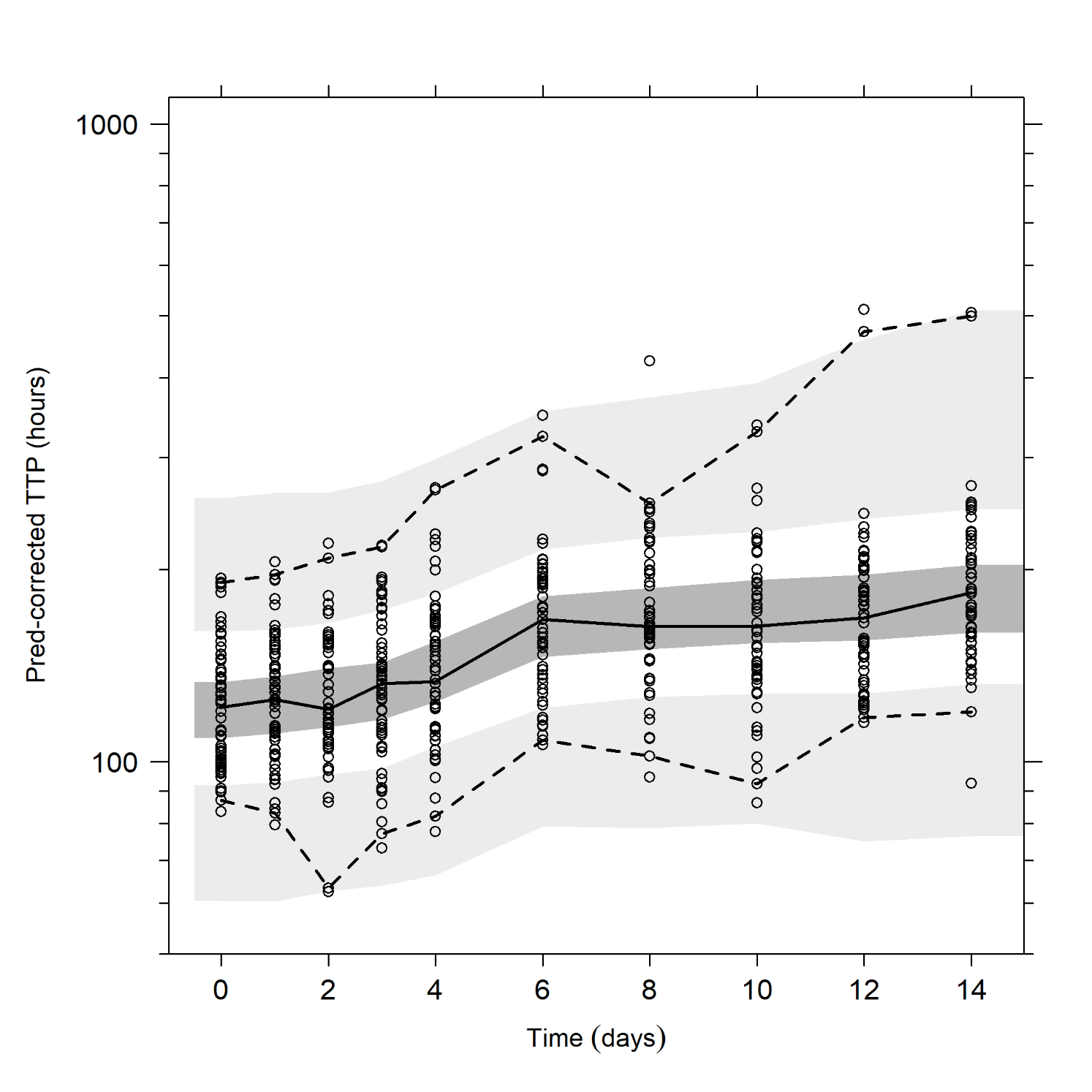
Figure S7. Illustration of a prediction-corrected visual predictive check (pcVPC) of the final early bactericidal activity time-to-positivity (EBA-TTP) model. The solid line represents the median prediction-corrected observation and the dark grey shaded area is the simulation-based 95% confidence interval for the median. The observed 5% and 95% percentiles are presented as dashed lines and the 95% confidence intervals for the corresponding model predicted percentiles are shown as light grey shaded areas. The open circles are the prediction-corrected observations. Plots are based on the simulated data to visualize the standardized pharmacometric model-based EBA analysis approach.
